# Audible acoustics from low-magnitude fluid-induced earthquakes in Finland

**DOI:** 10.1038/s41598-021-98701-6

**Published:** 2021-09-28

**Authors:** Oliver D. Lamb, Jonathan M. Lees, Peter E. Malin, Tero Saarno

**Affiliations:** 1 Department of Geological Sciences, University of North Carolina at Chapel Hill, Chapel Hill, NC USA; 2 Earth and Ocean Sciences, Nicholas School of the Environment, Duke University, Durham, NC USA; 3 ASIR Advanced Seismic Instrumentation and Research, Raleigh, NC USA; 4 St1 Deep Heat Oy, Helsinki, Finland

**Keywords:** Environmental sciences, Natural hazards, Geophysics, Seismology

## Abstract

Earthquakes are frequently accompanied by public reports of audible low-frequency noises. In 2018, public reports of booms or thunder-like noises were linked to induced earthquakes during an Engineered Geothermal System project in the Helsinki Metropolitan area. In response, two microphone arrays were deployed to record and study these acoustic signals while stimulation at the drill site continued. During the 11 day deployment, we find 39 earthquakes accompanied by possible atmospheric acoustic signals. Moment magnitudes of these events ranged from $-0.07$ to 1.87 with located depths of 4.8–6.5 km. Analysis of the largest event revealed a broadband frequency content, including in the audible range, and high apparent velocities across the arrays. We conclude that the audible noises were generated by local ground reverberation during the arrival of seismic body waves. The inclusion of acoustic monitoring at future geothermal development projects will be beneficial for studying seismic-to-acoustic coupling during sequences of induced earthquakes.

## Introduction

Earthquakes of a wide range of magnitudes are commonly accompanied by reports and/or measurements of atmospheric acoustic waves at various epicentral distances. These waves may have frequencies ranging from infrasonic ($<20 \,\hbox {Hz}$) up to and beyond the minimum limit of human hearing ability (20–70 Hz). Cases of the latter have been described as low rumbling sounds or booms^1^, and have been reported for shallow ($<2\, \hbox {km}$) earthquakes in the USA^2^ and France^3–5^. The event magnitudes associated with these sounds have been stated to be as low as $-2$ and $-0.7$, respectively. Audible noises are also frequently publicly reported for larger magnitude earthquakes, and accompanied by the frequent detection of infrasonic acoustic waves at large distances (up to 5300 km)^6–16^. Mapping of acoustic sources during and immediately after earthquakes has identified three sources of earthquake acoustic signals^17^: (1) ‘epicentral’ (i.e. seismic-to-acoustic coupling directly above or near the earthquake epicentre)^7,8^, (2) ‘local’ (i.e. generated by the passage of seismic waves near sensor located away from the epicentre)^6,18,19^ and (3) ‘secondary’ (i.e. generated by interaction of seismic waves with topographic features)^8,11,20,21^. Efficient coupling of seismo-acoustic energy into the atmosphere has been attributed to three parts of the wavefield spectrum^22,23^: vertically propagating homogenous body waves (particularly P- and SV-waves)^24^, inhomogeneous body waves (a.k.a. evanescent waves)^25^, and surface waves, or more specifically, leaky Rayleigh or Stonely waves^26^. Seismo-acoustic recordings of earthquake acoustic signals (audible and infrasonic) at near ($<25 \,\hbox {km}$) or epicentral distances are limited to only a few studies^4,21,24^. Here we describe a case study of local acoustic waves generated by earthquakes during a hydraulic stimulation project in Finland, one of the first documented recordings of acoustic signals from an induced earthquake sequence and are amongst the lowest magnitude events to be recorded.

## St1 Deep Heat Oy venture

**Figure 1:**
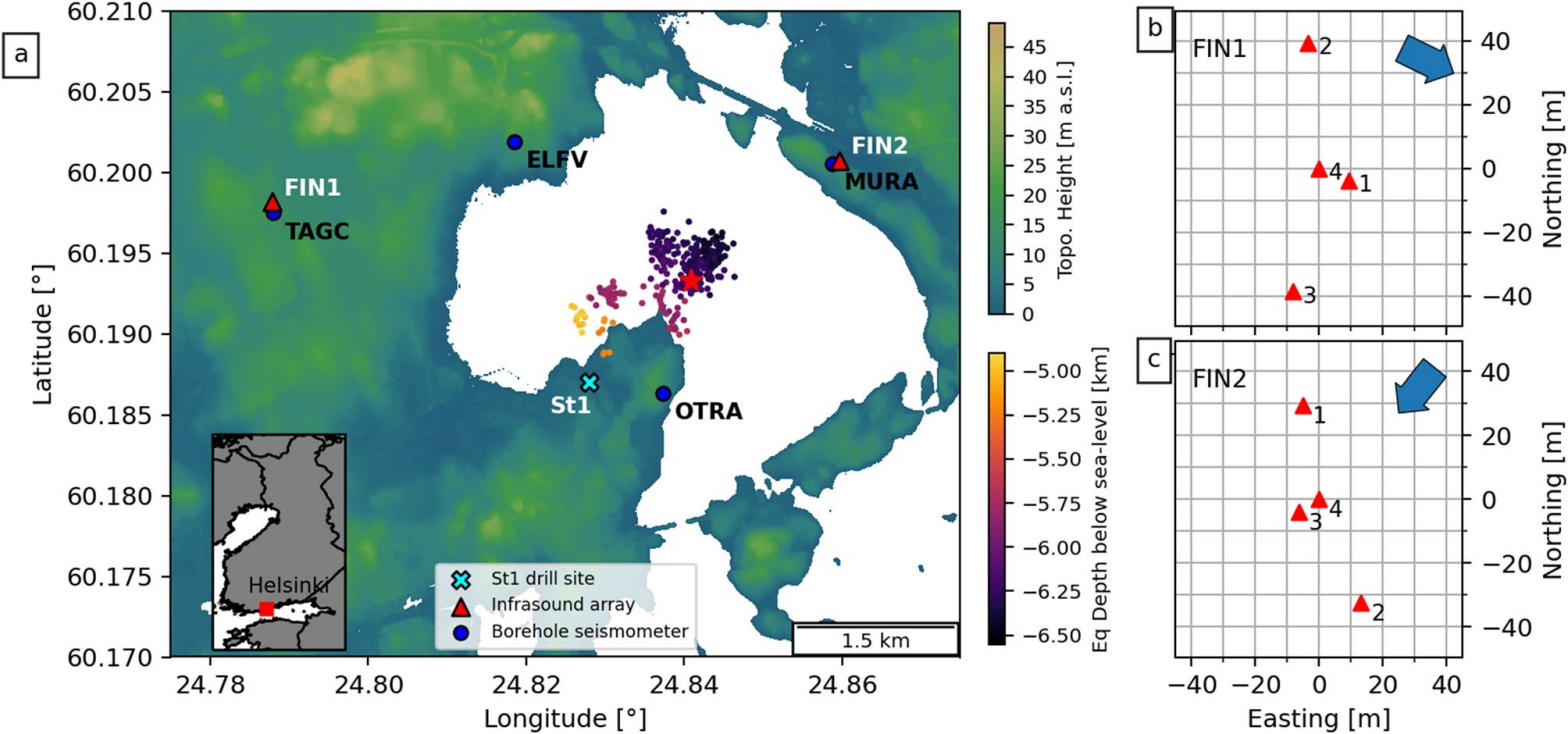
(**a**) Topographic map of the region around the St1 drill site (cyan cross) showing locations and names of borehole seismic stations (blue circles) and temporary acoustic arrays (red triangles). Also plotted are locations of earthquakes recorded during the acoustic deployment, colored by depth. Red star indicates the location of the $\hbox {M}_{\mathrm{w}}$ 1.87 event. Inset: Map of Finland showing location of the Helsinki Metropolitan area. Panels (**b**,**c**) show the infrasound sensor distribution for arrays FIN1 and FIN2, respectively, with back azimuth direction to the ST1 drill site indicated by the blue arrow. Topographic data used in panel (**a**) were downloaded from the National Land Survey of Finland via the Open data file download service (last accessed December 2020). This figure was generated using Matplotlib (v. 3.2.2; matplotlib.org)^31^ and Cartopy (v. 0.17.0; scitools.org.uk/cartopy)^32^.

The Engineered Geothermal System (EGS) pilot project, operated by the St1 Deep Heat Oy energy company, was located in the Helsinki Metropolitan area within the campus of Aalto University (Fig. 1). The aim of the project was to develop an EGS facility in order to produce a sustainable baseload for the local district heating system^27^. In 2018, a 6.1 km deep stimulation well was drilled into crystalline Precambrian Svecofennian basement rocks consisting of granites, pegmatites, gneisses, and amphibolites^27^. This bedrock features extensive faults, lineaments, and fractures^28^ and is only locally covered by a thin ($<10 \,\hbox {m}$) layer of glacial till or soil^29^. From 4 June to 22 July 2018, a total of 18,160 $\hbox {m}^{3}$ of water was pumped into the stimulation well at depths of 5.7–6.1 km; this included moving injection intervals and multiple stoppages for a few days^27,29^. Induced seismicity was monitored by an extensive seismic network, including 3-component borehole seismometers installed in 0.3–1.15 km deep wells at distances up to 8.2 km from the drill site (Fig. 1). The purpose of the seismic network was to provide accurate hypocenter locations and magnitudes of induced earthquakes for both industrial and regulatory purposes (i.e. Traffic Light System)^27,30^.

From 4 June to 1 August 2018, a total of 8412 earthquakes were automatically recorded by the network out of which 1977 were suitable for relocations and magnitude calculations^27^. These events were located across three distinct clusters ranging in depths of 4.8–6.6 km and moment magnitudes ($\hbox {M}_{\mathrm{w}}$) of $-0.76$ to 1.87 (Fig. S1 in Supporting Information). Fault plane solutions for a set of selected events indicated reverse faulting along pre-existing fractures associated with NW–SE trending fault zones reactivated by the hydraulic injection^29,33^. Propagation directions of SH waves across local seismic arrays show deviations from the earthquake back azimuths that may be related to the local heterogeneous seismic structure^34^. The Institute of Seismology at the University of Helsinki (ISUH) collected 220 public reports of felt earthquakes, which unexpectedly also included dozens of audible disturbances, typically described as thunder- or blast-like^29,30^. The largest and most reported event was a $\hbox {M}_{\mathrm{w}}$ 1.87 event on 8 July 2018 located at 6.3 km depth (Fig. 1). This event generated 78 public reports and was apparently heard up to 9 km away from the epicentre^29^. Notably, spatial distributions of the reports were strongly correlated with the SH radiation pattern of the reverse faulting mechanism in the event^29^.

## Data and methods

In response to the reports of audible earthquake events, we deployed two temporary arrays of infrasound microphones in the area from 7 to 18 July to study the nature of these atmospheric acoustic signals. The arrays were deployed at distances of $\sim \,2.5$ and $\sim \,2.2 \,\hbox {km}$ from the St1 drill site. Each deployment consisted of three microphones extended on cables up to 35 m from a central data recorder, where a fourth microphone was located (Fig. 1b,c). The data recorder was a REFTEK RT 130 data logger which provided a 24-bit, GPS-time synchronized recording set to 100 samples per second, resulting in an anti-aliasing Finite Impulse Response (FIR) filter cut off of 40 Hz. The microphones were identical InfraBSU (vers1) microphones, which incorporate a MEMS sensor and capillary filters to provide a flat response from 0.1 up to $>40 \,\hbox {Hz}$^35^. To aid analysis and interpretation of acoustic data in this study, we also included seismic data from borehole seismometers located near each array (TAGC and MURA; Fig. 1a). Each seismometer was composed of a three-component Sunfull PSH geophone sensor ($\textit{f}_{\mathrm{N}} = 4.5 \,\hbox {Hz}$) recording at 500 samples per second and located $\sim 1.15 \,\hbox {km}$ below the surface (for more information, see Kwiatek et al.^27^).

For this study, all data were filtered with a 2 Hz high-pass Butterworth filter to reduce continuous background noise (unless otherwise indicated). Data were manually inspected for consistent arrivals across at least two microphones in each array to assess if earthquake-generated atmospheric acoustic waves were detected following an induced earthquake. To estimate the arrival times for different body wave phases at each array, we use P- and S-wave velocities of 6.25 and $3.75 \,\hbox {km s}^{-1}$ respectively, as estimated from borehole logs at the St1 drill site (see supplementary materials in Kwiatek et al.^27^). One of the key advantages of deploying acoustic microphones in an array configuration is it permits the calculation of back azimuth direction and apparent velocities of acoustic waves propagating across the deployment. Back azimuth is calculated using least-squares beamforming where time delays between sensors are calculated using cross-correlation^36^. Here we estimated back azimuths and apparent velocity values for 0.5 s windows with 90% overlap within the first 3 s after the initiation time of the earthquake. Windows in which calculated apparent velocity were below physically possible values (i.e. $<0.25\, \hbox {km s}^{-1}$) or relative power was lower than 0.6 were discarded. Relative power is defined as the signal power of the mean waveform for minimum apparent velocity divided by average element power in the same time window. We find that waveforms tend to lack coherency between sensors, therefore we used waveform envelopes, determined from the square root of the Hilbert Transform, which were then smoothed using the average of an 8 sample moving window (Fig. 4a,b). All analysis presented here was carried out within the ObsPy python package^37^.

## Observations

**Figure 2:**
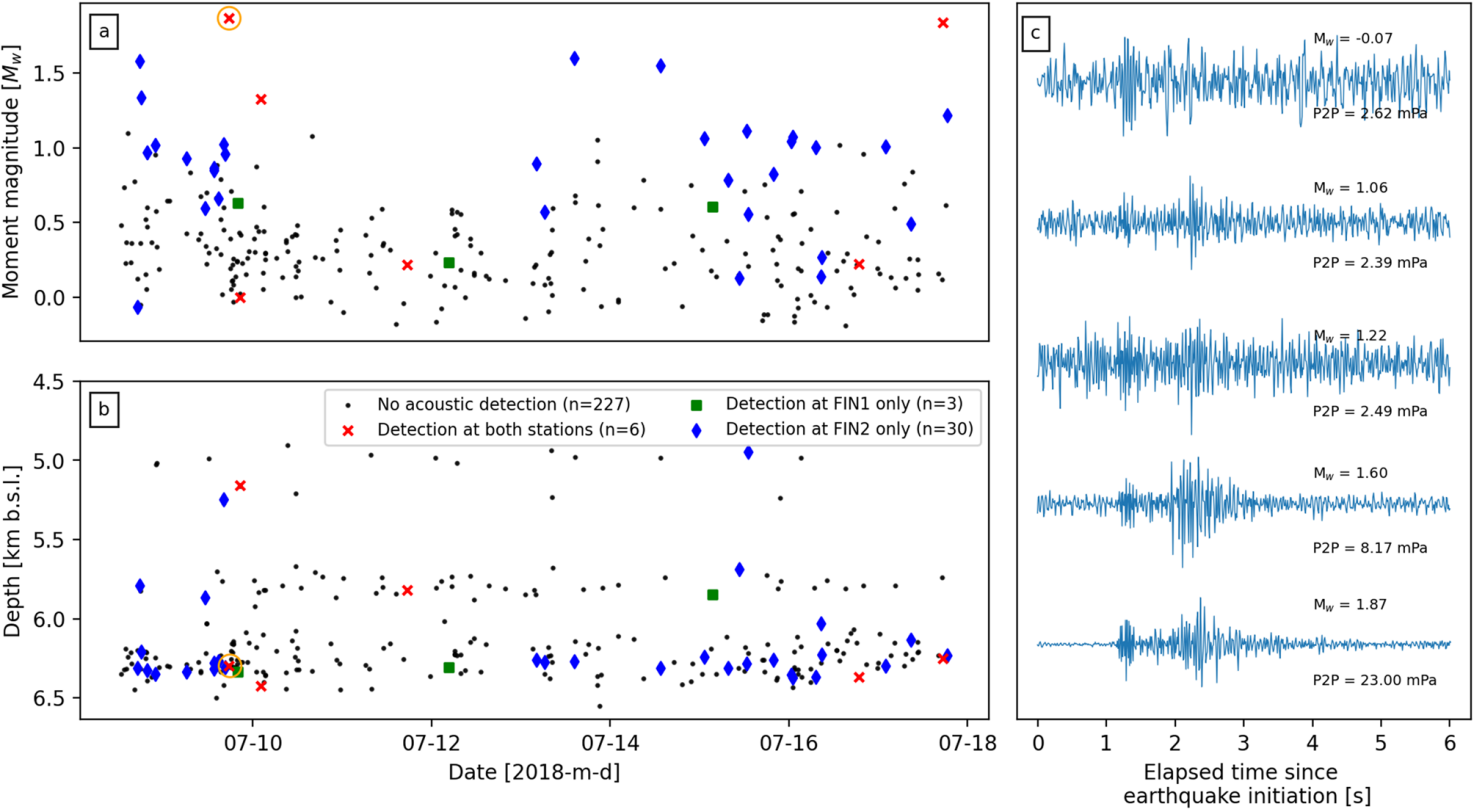
Moment magnitudes (**a**) and depths (**b**) of the 266 relocated seismic events recorded during the infrasound array deployment near the St1 Deep Heat Oy EGS project. Red ‘x’, green squares, and blue diamonds indicate the events which were detected by both acoustic arrays, only at FIN1, or only at FIN2, respectively. Orange circle in each panel indicates the $\hbox {M}_{\mathrm{w}}$ 1.87 event described in detail in Figs. 3 and  4. (**c**) 6 s of normalised acoustic data (highpass filtered at 5 Hz) recorded by sensor 2 at FIN2 after the initiation of five example earthquakes, including the lowest and highest magnitude events. Calculated $\hbox {M}_{\mathrm{w}}$ and recorded peak-to-peak pressure amplitudes (P2P) of each event is indicated on the right; each event was located at 6.2–6.3 km depth (see Figs. S2 to S11 in Supporting Information for waveforms and frequency spectrograms from all microphones for each event).

During 7–18 July, 266 earthquakes were detected and relocated within a few hundred metres of the stimulation interval. These events occurred at depths of 4.8–6.5 km below sea level and had moment magnitudes ranging from $-0.19$ to 1.87 (Figs. 1a, 2a,b). Through manual inspection of the acoustic data, 39 of the 266 earthquakes were followed shortly by possible atmospheric disturbances across at least one array that may be interpreted as earthquake associated acoustic waves (Fig. 2). Atmospheric disturbances were more commonly seen at FIN2 ($\hbox {n}=36$) than FIN1 ($\hbox {n}=9$), with only 3 events seen exclusively at the latter. The smallest event was a $\hbox {M}_{\mathrm{w}}$ $-0.07$ on 8 July, and the largest was the widely heard $\hbox {M}_{\mathrm{w}}$ 1.87 on the same day (Fig. 2c). As the latter earthquake produced the highest signal-to-noise ratios at both microphone arrays, the remainder of this section will focus on the analysis of acoustic data from this particular event. Similar analysis as below has been conducted on the other four example events in Fig. 2c and detailed in Figs. S12 to S15 in Supporting Information. We find that the acoustics recorded shortly after the $\hbox {M}_{\mathrm{w}}$ 1.87 contain the only waveforms that can be confidently attributed to the earthquake due to the back azimuth and apparent velocity calculations.

**Figure 3:**
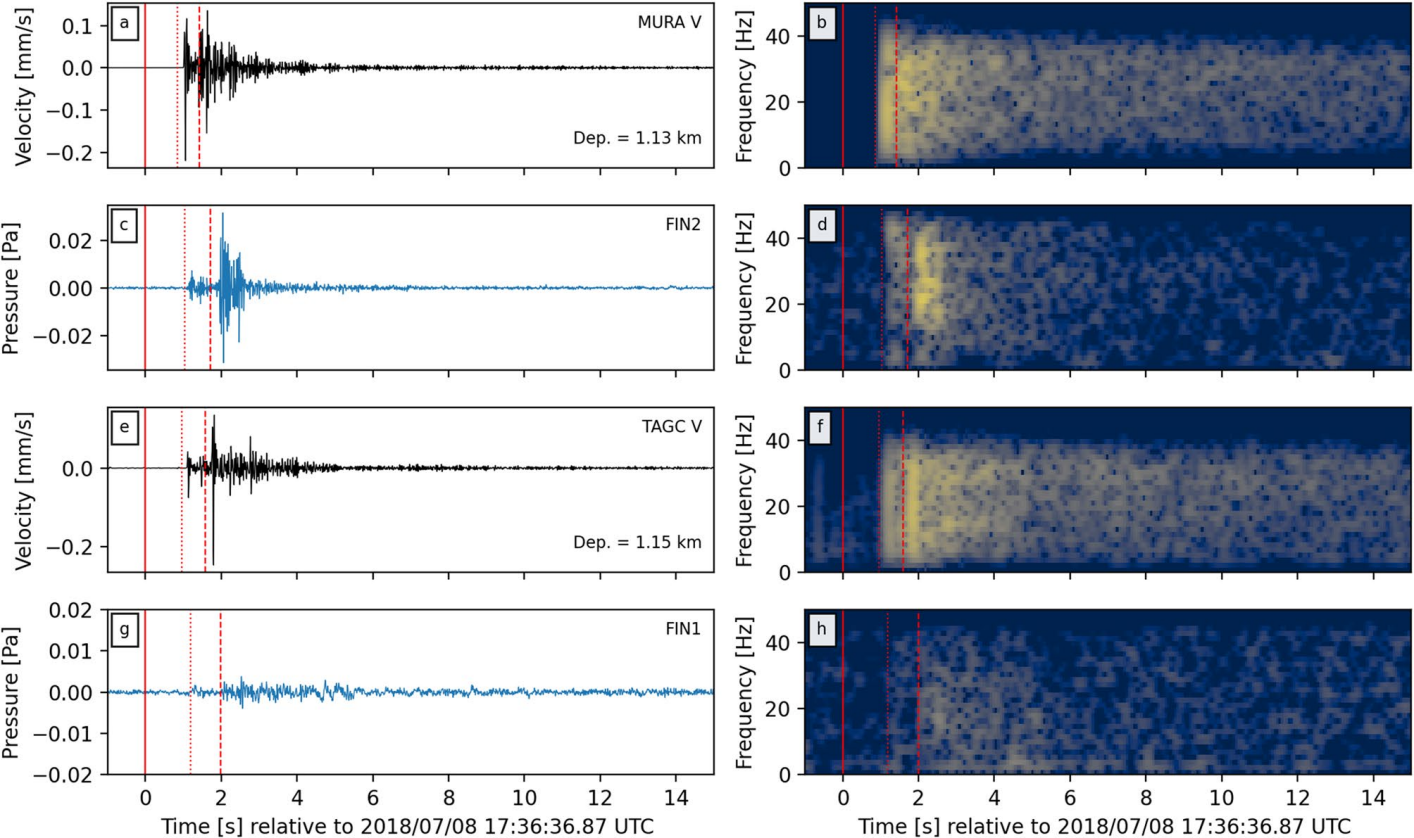
Filtered waveforms (left column) and their respective frequency spectrograms (right column) of the $\hbox {M}_{\mathrm{w}}$1.87 event as recorded by seismic station MURA (**a**,**b**), acoustic array FIN2 (**c**,**d**), seismic station TAGC (**e**,**f**) and acoustic array FIN1 (**g**,**h**). Note that the seismic waveforms are from the vertical component of the station. Spectrograms were calculated with 0.5 s windows with 90% overlap. Also plotted is the time of the event (solid red line), as well as predicted arrival times for P- and S-wave phases (dotted and dashed red lines, respectively) from source locations to each station or array.

**Figure 4:**
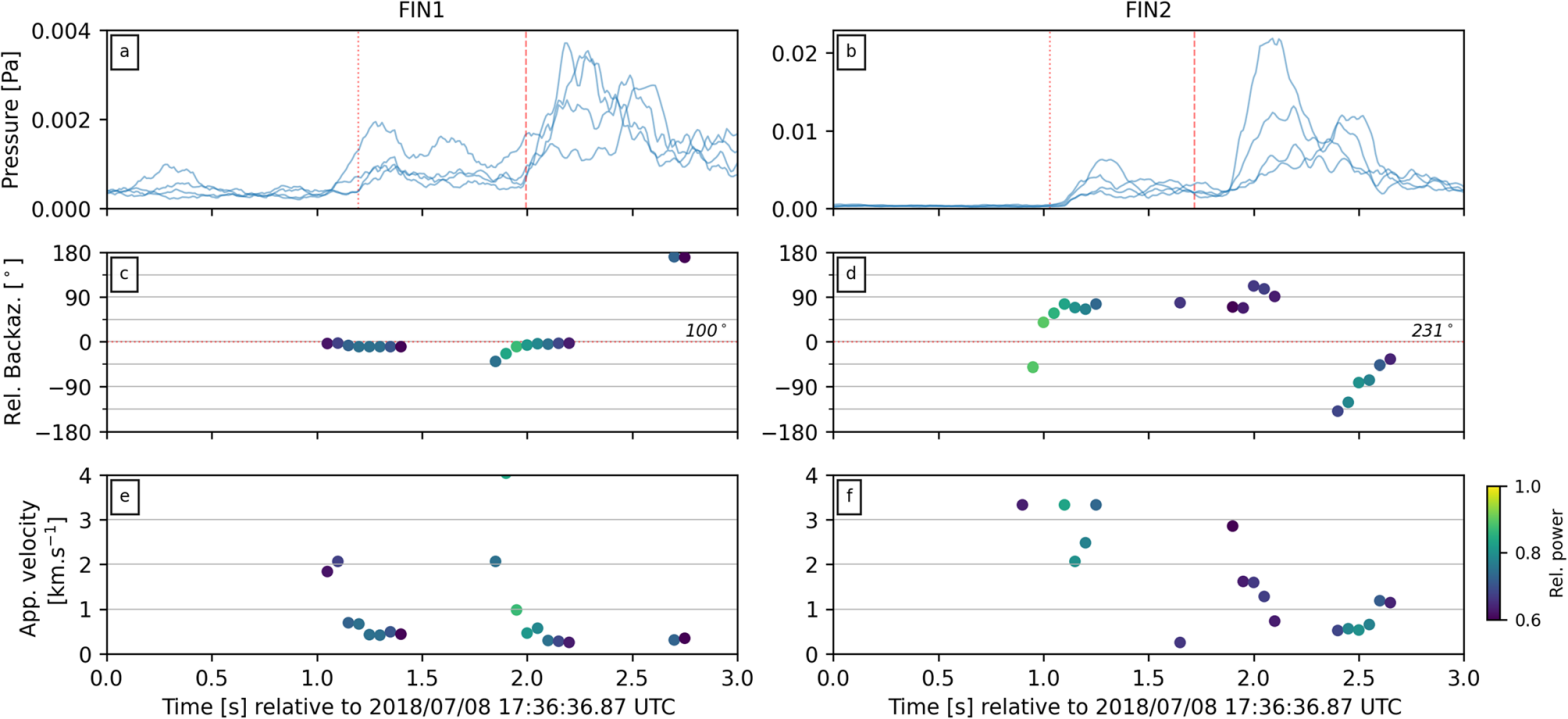
Beamforming results for arrays FIN1 (left column) and FIN2 (right column) for the first 3 seconds after the $\hbox {M}_{\mathrm{w}}$1.87 event. (**a**,**b**) Smoothed waveform envelopes from each element in each array. Dotted and dashed lines plot the estimated arrival times of P- and S-waves, respectively (from epicentre to array). (**c**,**d**) Back azimuth calculations for 0.5 s moving windows with 90% overlap, relative to the theoretical back azimuth from array to the $\hbox {M}_{\mathrm{w}}$1.87 event epicentre (horizontal dotted red line, absolute back azimuth value labeled on right hand side). (**e**,**f**) Calculated apparent velocity values across each array for each 0.5 s window. Points in panels (**c**–**f**) are colored by relative power, where lighter colors indicate higher relative power.

For the $\hbox {M}_{\mathrm{w}}$ 1.87 event the acoustic data recorded at FIN2 have peak amplitudes an order of magnitude larger than those recorded at FIN1 (Fig. 3c,g). Frequency spectra highlight the broadband nature of the atmospheric acoustic signals, with frequencies ranging from 2 to 40 Hz (Fig. 3d,h), which are the limits set by the filter and sampling rates (see Data and Methods section). The acoustic waves and their spectra at each array appear to show distinct multi-phase arrivals that correlate with seismic waves recorded at the nearby borehole seismometers (Fig. 3a,b,e,f). The different arrival phases at each array appear to be coincident with the predicted arrivals of P- and S-waves (dotted and dashed red lines in Fig. 3). The highest acoustic amplitudes are correlated with the arrival of the S-waves at each array with time offsets between acoustic and seismic arrivals correlating with depths of seismic stations (noted in panels a and e of Fig. 3). Calculated values of back azimuth and apparent velocities at or near the estimated time of arrivals for P- and S-waves (red lines in Fig. 4a,b) indicate arrivals from the direction of the $\hbox {M}_{\mathrm{w}}$ 1.87 event epicentre (Fig. 4c,d). Apparent velocity values at these times indicate relatively initially high propagations across the array, which rapidly decrease to lower values in the subsequent time windows (Fig. 4e,f). For comparison, similar analysis was conducted on a $\hbox {M}_{\mathrm{w}}$ 1.84 event that occurred on July 16. Despite clear waveforms arriving at each array (Figs. S12, S13 in Supporting Information), the back azimuth and apparent velocities across the arrays did not correlate with expected values from the event (Fig. S18 in Supporting Information).

## Discussion

Here we have presented evidence for infrasonic and audible atmospheric acoustic signals generated by at least one low magnitude fluid-induced earthquake. These observations are notable for two reasons: (1) these are the first recorded earthquake-generated acoustic signals from induced earthquakes, and (2) they represent the lowest magnitude events to be recorded by acoustic microphones. (There are reports of audible noises from earthquakes with magnitudes as low as $-2$ but these events were not recorded with microphones^5^.) Manual inspection of data identified at least 39 events where possible acoustic waves were recorded propagating across at least one array of sensors (Fig. 2). This represents only 15% of all earthquakes relocated during the deployment, but the location of the arrays within a large metropolitan area with a large number of low-frequency noise sources may have acted to reduce this proportion. On the other hand, the potential for false associations due to coincidental sound arrivals from anthropogenic sources would suggest this 15% value may be an overestimate. Further analysis of the waveforms across each array finds that only the acoustics shortly after largest earthquake can be reasonably attributed as having derived from the seismic event. The acoustic waves contained broadband frequency ranges from 2 up to 40 Hz, and possibly higher but is limited by the anti-alias FIR filter of the sample recording rate (Fig. 3d,h). This broadband frequency is often but not always observed for other possible acoustic signals from earthquakes (Figs. S2–S13 in Supporting Information) and this is most likely due to low signal-to-noise ratios. Nevertheless, this frequency range overlaps with the lower range of human hearing (down to 20 Hz), therefore supporting the notion that thunder- or blast-like sounds heard by the public were generated by the earthquakes^29,30^. These frequency ranges also match previously reported values from audible natural earthquakes^4,24^.

Given that the infrasound sensors are typically placed in direct contact with the ground surface during deployments, contamination of recorded infrasound signals by physical shaking of the sensor could be a concern. However, testing of the seismic response of various acoustic sensors have consistently concluded that physical vibration does not significantly influence the recorded infrasound signals^4,24,38^. The MEMS-based microphones used in this study (InfraBSU vers1) have low inertial mass and are similar in design to the MEMS-based transducers described in Marcillo et al.^35^. These sensors were found to have minimal seismic-to-noise coupling during calibration studies at the Facility for Acceptance, Calibration and Testing site at the Sandia National Laboratories^21^. Therefore, we do not consider direct seismic shaking of the sensor to be of importance in the acoustic signals presented here.

During the expected arrival times of the P- and S-waves for the $\hbox {M}_{\mathrm{w}}$1.87 event at each array, the back azimuth values align at or around the direction of the earthquake epicentre (Fig. 4c,d). It is notable that a significant number of windows were discarded due to unrealistic apparent velocity values or low relative power. Furthermore, similar calculations for acoustic waveforms from other events produced poor or inconclusive results (Fig. S14–S18 in Supporting Information). This is likely due to low signal-to-noise ratios, the low sampling rates chosen (100 samples per second), poor array-perpendicular apparent velocity resolution due to the narrow deployment configuration of the arrays, or technical issues with individual sensors. Ideally, 3 or 4 microphone sensor arrays would be arranged as an equilateral triangle. However, the geometry of each array here was forced by the limited availability of deployment areas which is to be expected for a rapid response deployment in an urban environment. Nevertheless, azimuthal resolution is expected to be good and poor for bearings perpendicular and parallel to the arrays, respectively. Calculated infrasound array uncertainties following the method of Szuberla and Olson^39^ indicate a minimum uncertainty for back azimuth of $10^{\circ }$ for each array given a 95% confidence interval (Figs. S19, S20 in Supporting Information). The consistent deviation between calculated back azimuths and great-circle direction to the earthquake epicentre at FIN2 (Fig. 4d) may be related to either: (1) the non-optimal array configuration or (2) the locally heterogeneous seismic structure^28^. The latter was inferred to explain similar deviations at local seismic arrays deployed in the same region during the same induced seismic sequence^34^.

A common observation in previous earthquake acoustic studies is the presence of ‘local’ infrasound at the sensor location^6,18,19^, as well as ‘secondary infrasound’ generated away from the earthquake epicentre^8,10,11,17,20,21^. Calculated apparent velocity values during the arrival of seismic waves from the $\hbox {M}_{\mathrm{w}}$ 1.87 event begin with relatively high propagation velocities across the array, but rapidly decrease to lower values (Fig. 4e,f). The initially high velocities ($>1 \,\hbox {km s}^{-1}$) suggest the presence of ‘local’ infrasound generated during the passage of seismic waves across the array, but could also indicate near-vertical wave arrival directions at the array. Considering the ratio between earthquake depths (4.8–6.5 km) and epicentre-array distances ($< 2.5 \,\hbox {km}$), it is reasonable to expect near vertical arrival angles of seismic waves at each array. The relationship between vertical ground motion to air pressure has been formulated as $\Delta P=\rho cv$ where $\rho$ is the density of air ($1.225 \,\hbox {kg m}^{-3}$), *c* is the velocity of sound in the air ($330 \,\hbox {m s}^{-1}$), *v* is the vertical ground velocity, and $\Delta P$ is the measured pressure fluctuation^6,18,19^. At station MURA, the peak vertical velocity during the P-wave arrival was $0.2 \,\hbox {mm s}^{-1}$ which, according to the above equation, should correspond to a 0.08 Pa acoustic pressure wave. This significantly overestimates what was measured at the surface at station FIN2 during the arrival of P-waves ($<0.01 \,\hbox {Pa}$; Fig. 3). This may be due to attenuation of the seismic waves between MURA (at 1.13 km depth) and the surface, unquantified local site effects at each station, or this back-of-the-envelope calculation is too simplistic to quantify local seismic-to-acoustic coupling for low magnitude events.

The lower propagation velocities are of the same magnitude as atmospheric acoustic waves ($\sim 330 \,\hbox {m s}^{-1}$). This can be interpreted as ‘secondary infrasound’ from sources in close proximity to the arrays ($<150 \,\hbox {m}$), within the same back azimuth from source to receiver. These acoustic signals are confirmed to be caused by the interaction of surface waves with topography or other significant crustal features^11,17^. Considering the lack of steep topographical features around the St1 drill site (Fig. 1a), it’s possible the secondary acoustic signals were instead generated by mechanical shaking of buildings or other structures (e.g. bridges) near each array. However, it is worth noting that velocity resolution perpendicular to the arrays is poor due to the forced narrow deployment configuration (Figs. S16, S17 in Supporting Information).

‘Epicentral’ infrasound is not considered a significant source of acoustics during these earthquakes due to the epicentre-station distances. For example, the epicentre of the $\hbox {M}_{\mathrm{w}}$1.87 event was 4.1 and 1.3 km from FIN1 and FIN2, respectively. Assuming an atmospheric acoustic velocity of $330\, \hbox {m s}^{-1}$, we would estimate an arrival time of approximately 12.4 and 3.9 s for ‘epicentral’ infrasound at FIN1 and FIN2. No clear arrival signals at these times are seen in the recorded waveforms (Fig. 3c,g). Furthermore, the epicentres have been located at 4.8–6.5 km depth beneath a shallow lagoon (Fig. 1a). Theoretical studies have shown that acoustic radiation into the atmosphere at water-gas or solid-gas interfaces may only be detectable when the earthquake is located at a depth on the order of the wavelength or less^22,25^. Therefore, the atmospheric acoustic signals recorded during the largest earthquake, and all other recorded events, were likely generated by ground motion at and near the station during and immediately after the arrival of P- and S-waves at the ground surface within close proximity of the microphone arrays. In other words, we record both ‘local’ and ‘secondary’ infrasound during the passage of seismic waves across the microphone arrays during the sequence of induced earthquakes.

A notable observation from the public reports compiled during the induced earthquake sequence is the geographical distribution of disturbances correlated with the radiation patterns of S-waves (see Fig. 5 in Hillers et al.^29^). The FIN2 acoustic array was located adjacent to the area with the greatest number of reports. This pattern correlates with the amplitude difference between the acoustic waves recorded at FIN1 and FIN2 for the $\hbox {M}_{\mathrm{w}}$ 1.87 event, with amplitudes an order of magnitude higher at the latter than the former (Fig. 3c,d). Furthermore, a higher number of apparent earthquake-generated acoustic waves were recorded at FIN2 (N = 36) than at FIN1 (N = 9). Another factor to consider is that the FIN1 array was deployed on the margin of an active golf course which was built on top of a former municipal waste landfill, while FIN2 was deployed within a small forested locality near an area where buildings are frequently constructed directly onto outcropping bedrock. This suggests that the presence of a soft sedimentary layer above the bedrock may act as a dampener during seismic-to-acoustic coupling of body waves. Previous observations have suggested that low frequency ($<10 \,\hbox {Hz}$) signals in the coda of acoustic waves may be generated by Rayleigh waves in a thin ($<100 \,\hbox {m}$) sedimentary layer above the bedrock^4^. No such low frequency coda is evident in the recordings seen here (Fig. 3d,h). This also contradicts observations from fast field program modeling at the ground-atmosphere interface that suggested enhanced seismo-acoustic coupling due to the presence of a sedimentary layer^23^. The observations described here suggests further work may be needed to test for seismo-acoustic coupling effects during a range of various sedimentary layer properties (e.g. thicknesses, small versus large basins, poorly consolidated versus well consolidated sediment). Nevertheless, the correlation between public sound report distributions and the acoustic amplitudes highlights the potential utility of such reports for monitoring at future EGS projects, particularly when high-quality geophysical recordings may not be available.

## Conclusions

Acoustic monitoring can help explain human observations and may also provide quantitative insights into the mechanics of ground motions responsible for generating earthquake sounds. Here we have presented acoustic events recorded within the Helsinki Metropolitan area in July 2018 during hydraulic stimulation at a pilot Engineered Geothermal System project. Based on the estimated timing of body wave arrivals, frequency content of the waveforms, as well as estimated apparent velocity calculations, we have interpreted these acoustic events as possibly being generated by reverberation of the ground surface during the arrival of P- and S-waves from induced low magnitude earthquakes. Although only a minor proportion of induced earthquakes generated recognizable acoustic waves, events with moment magnitudes ranging from $-0.07$ to 1.87 were recorded with acoustic microphones at the surface. Of these, only the largest event could be confidently attributed as having generated acoustic waves during the passage of seismic waves at each array. These events likely represent the first documented atmospheric acoustics of induced earthquakes and are amongst the lowest magnitude seismic events to be recorded with acoustic microphones. Given that Traffic Light Systems are increasingly being implemented to reduce the potential seismic hazard due to induced seismicity^30^, and the considerable public interest generated by audible earthquakes in the Helsinki Metropolitan area^29,30^, future projects for developing geothermal systems can benefit from deploying acoustic sensors to provide more detailed information in responses to public concern.

## Supplementary Information

Supplementary Information.
