# Supplementary material for: Audible acoustics from low-magnitude fluid-induced earthquakes in Finland
Source: Sci Rep. 2021 Sep 28;11:19206. doi: 10.1038/s41598-021-98701-6 (PMC8478913; doi:10.1038/s41598-021-98701-6)
Supplement: Supplementary file 1 — Supplementary Information. [file 41598_2021_98701_MOESM1_ESM.pdf]

Supporting Information for "Audible acoustics from low-magnitude fluid-induced earthquakes in Finland"

Oliver D. Lamb[1], Jonathan M. Lees[1], Peter E. Malin[2,3], Tero Saarno[4]

[1]Department of Geological Sciences, University of North Carolina at Chapel Hill, Chapel Hill, NC, USA

[2]Earth and Ocean Sciences, Nicholas School of the Environment, Duke University, Durham, NC, USA

[3]ASIR Advanced Seismic Instrumentation and Research, Dallas, TX, USA

[4]St1 Deep Heat Oy, Helsinki, Finland

Contents of this file

1. Figures S1 to S20

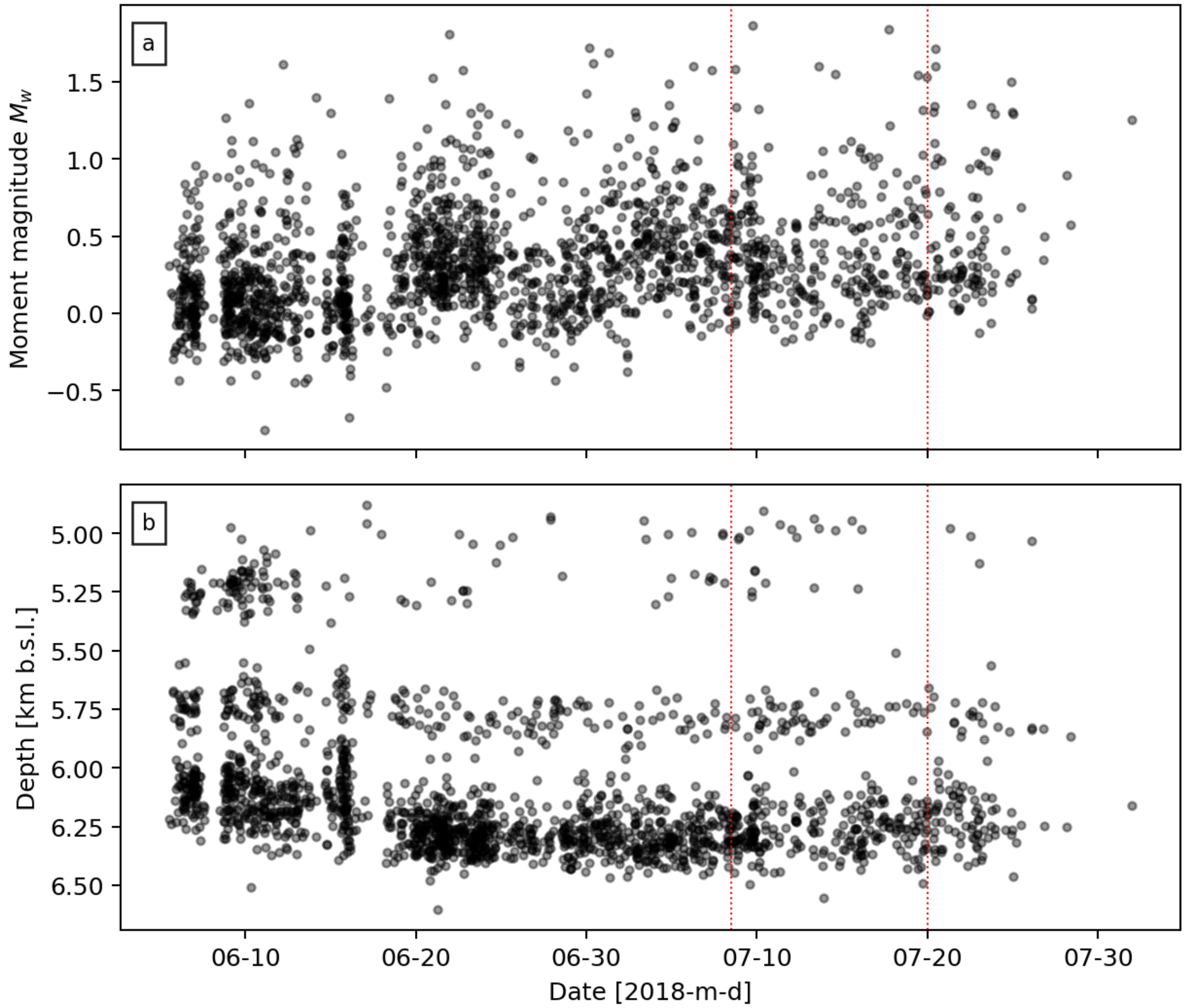

Figure S1. Details of 1977 earthquakes relocated during the hydraulic stimulation at the St1 Deep Heat Oy project. Vertical red dashed lines indicate the time period in which the microphones at FIN1 and FIN2 were deployed.

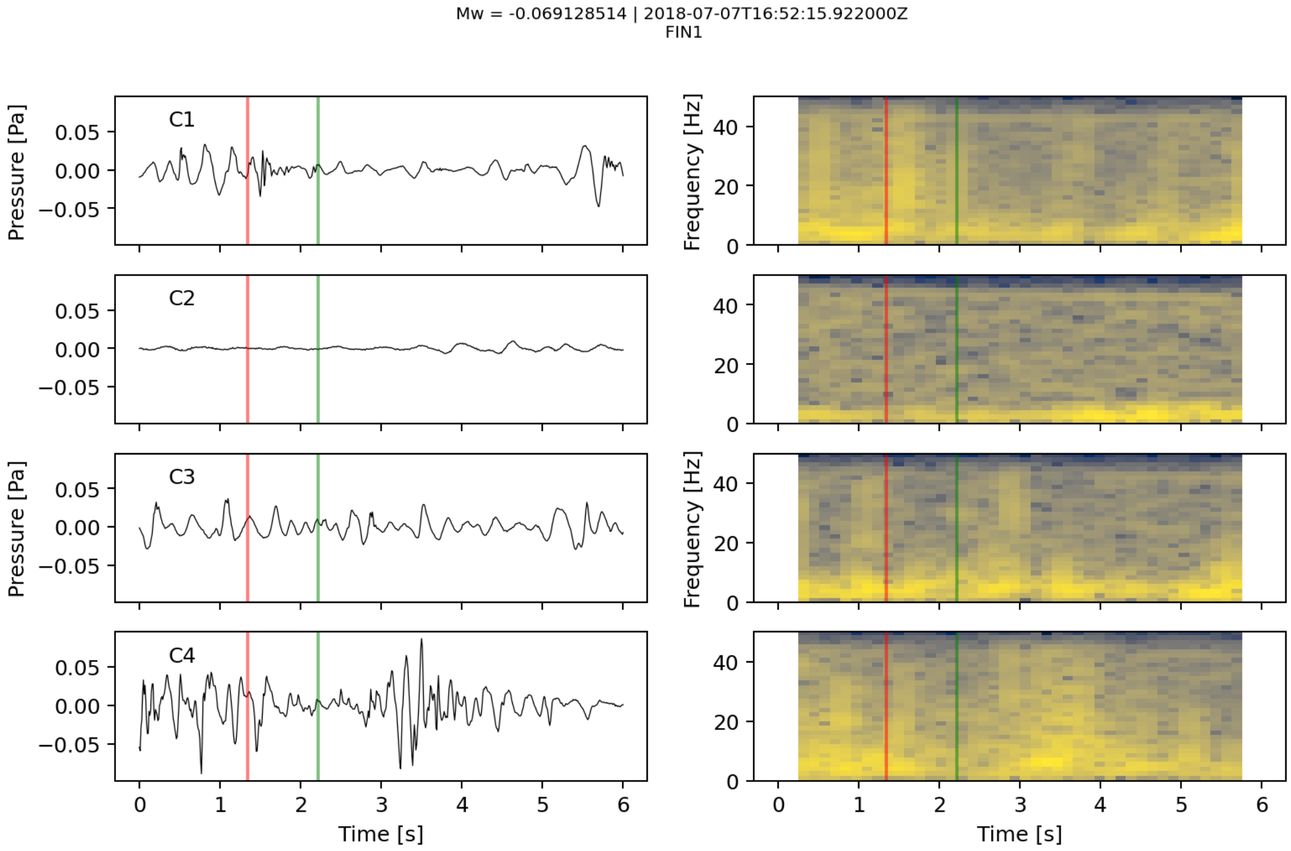

Figure S2. Waveforms (left column) and frequency spectrograms (right column) from FIN1 during a Mw -0.07 event. Red and green lines indicate estimated arrival times for P- and S-waves, respectively.

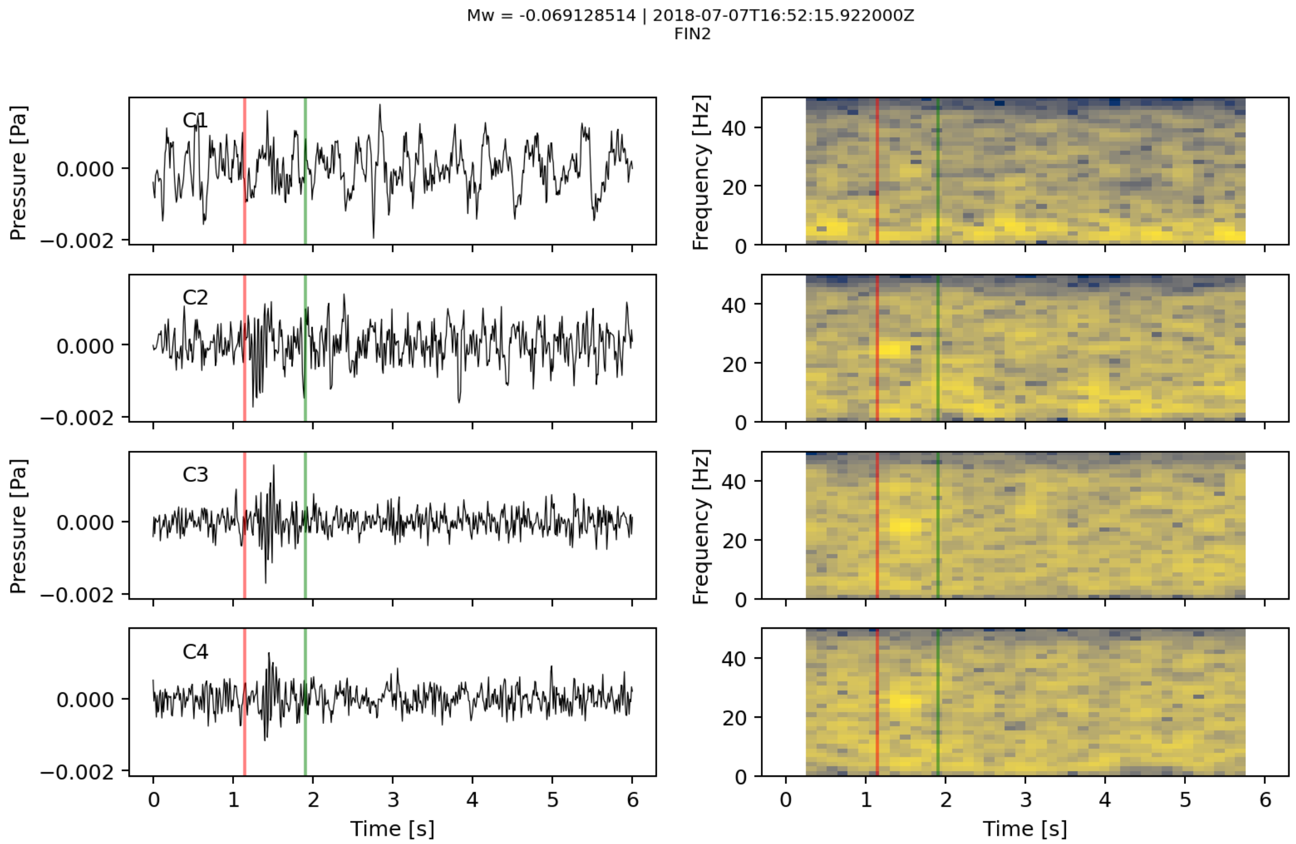

Figure S3. Waveforms (left column) and frequency spectrograms (right column) from FIN2 during a Mw -0.07 event. Red and green lines indicate estimated arrival times for P- and S-waves, respectively.

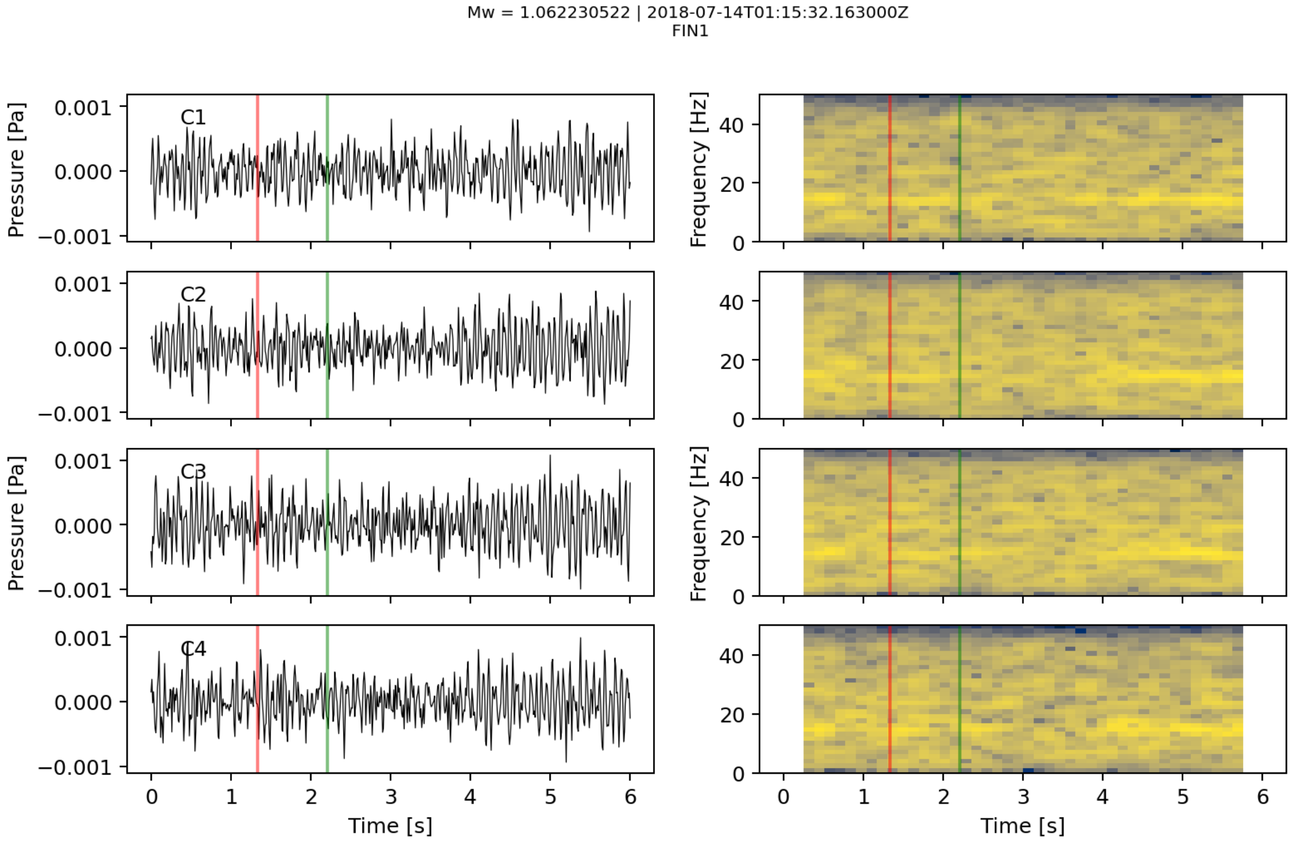

Figure S4. Waveforms (left column) and frequency spectrograms (right column) from FIN1 during a Mw 1.06 event. Red and green lines indicate estimated arrival times for P- and S-waves, respectively.

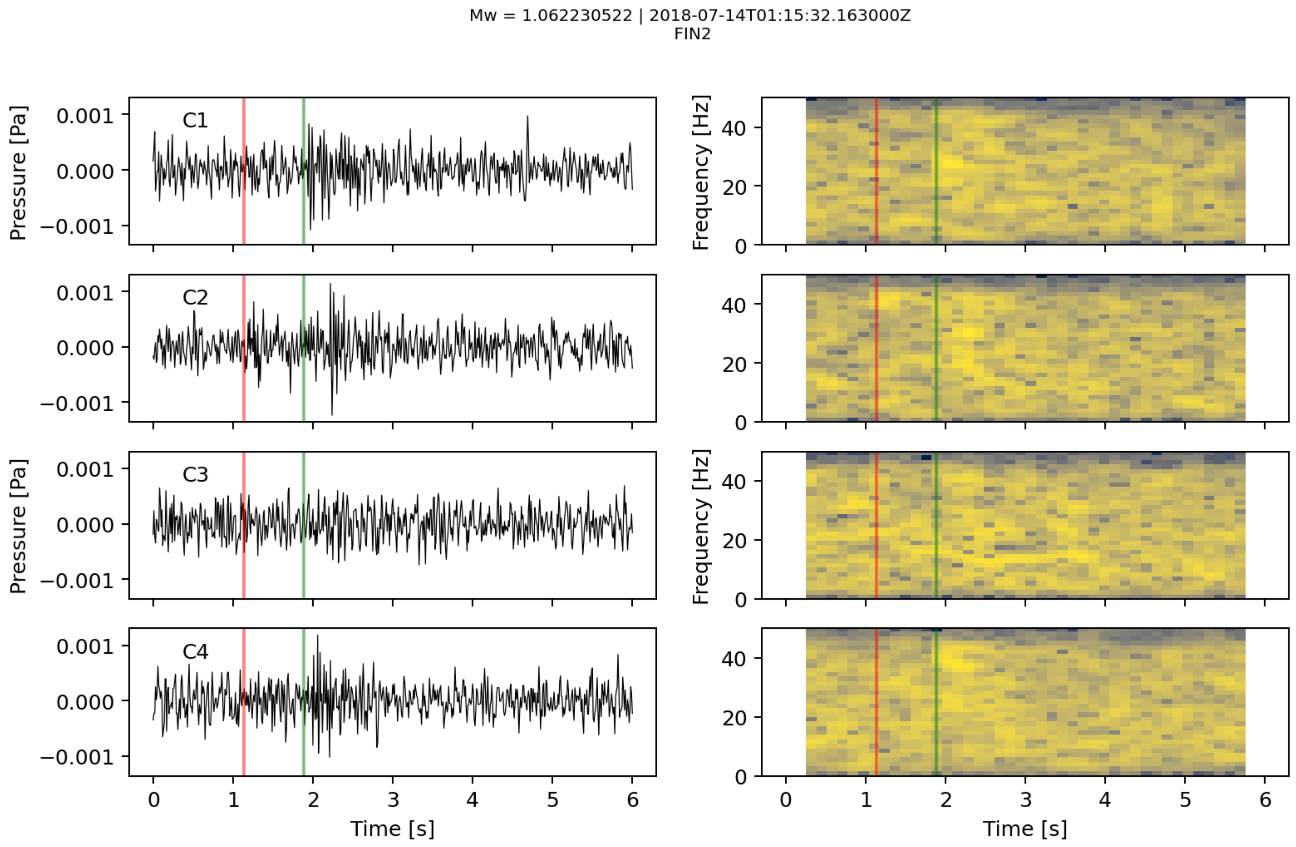

Figure S5. Waveforms (left column) and frequency spectrograms (right column) from FIN2 during a Mw 1.06 event. Red and green lines indicate estimated arrival times for P- and S-waves, respectively.

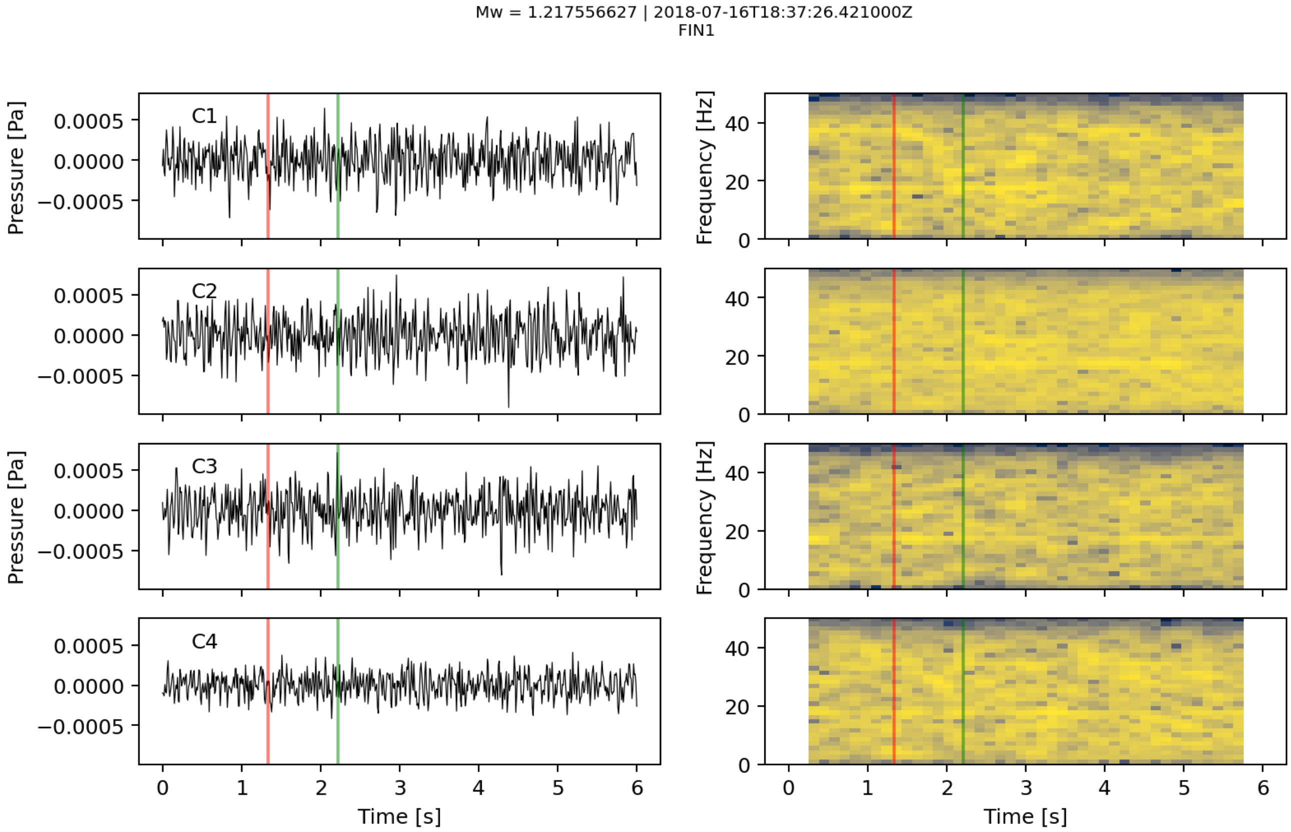

Figure S6. Waveforms (left column) and frequency spectrograms (right column) from FIN1 during a Mw 1.22 event. Red and green lines indicate estimated arrival times for P- and S-waves, respectively.

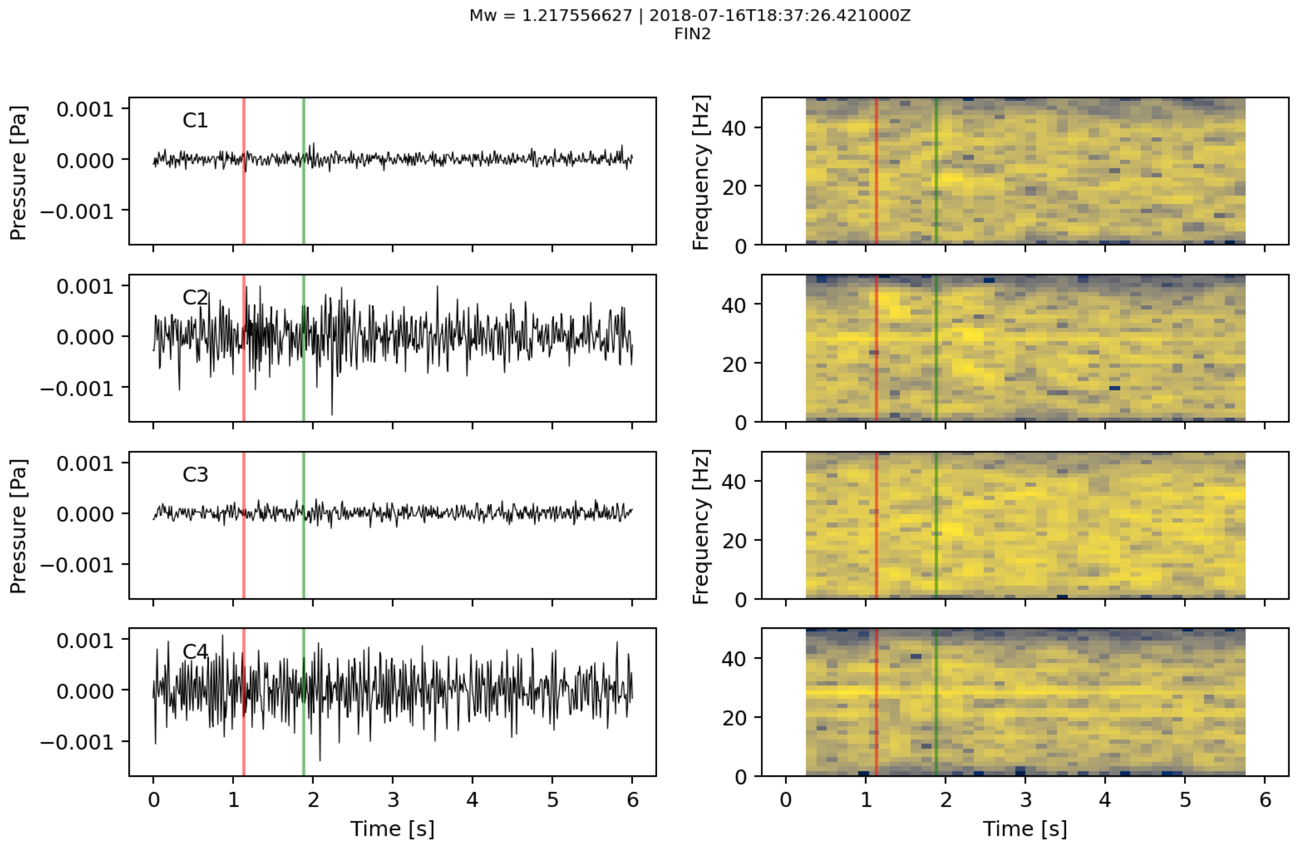

Figure S7. Waveforms (left column) and frequency spectrograms (right column) from FIN2 during a Mw 1.22 event. Red and green lines indicate estimated arrival times for P- and S-waves, respectively.

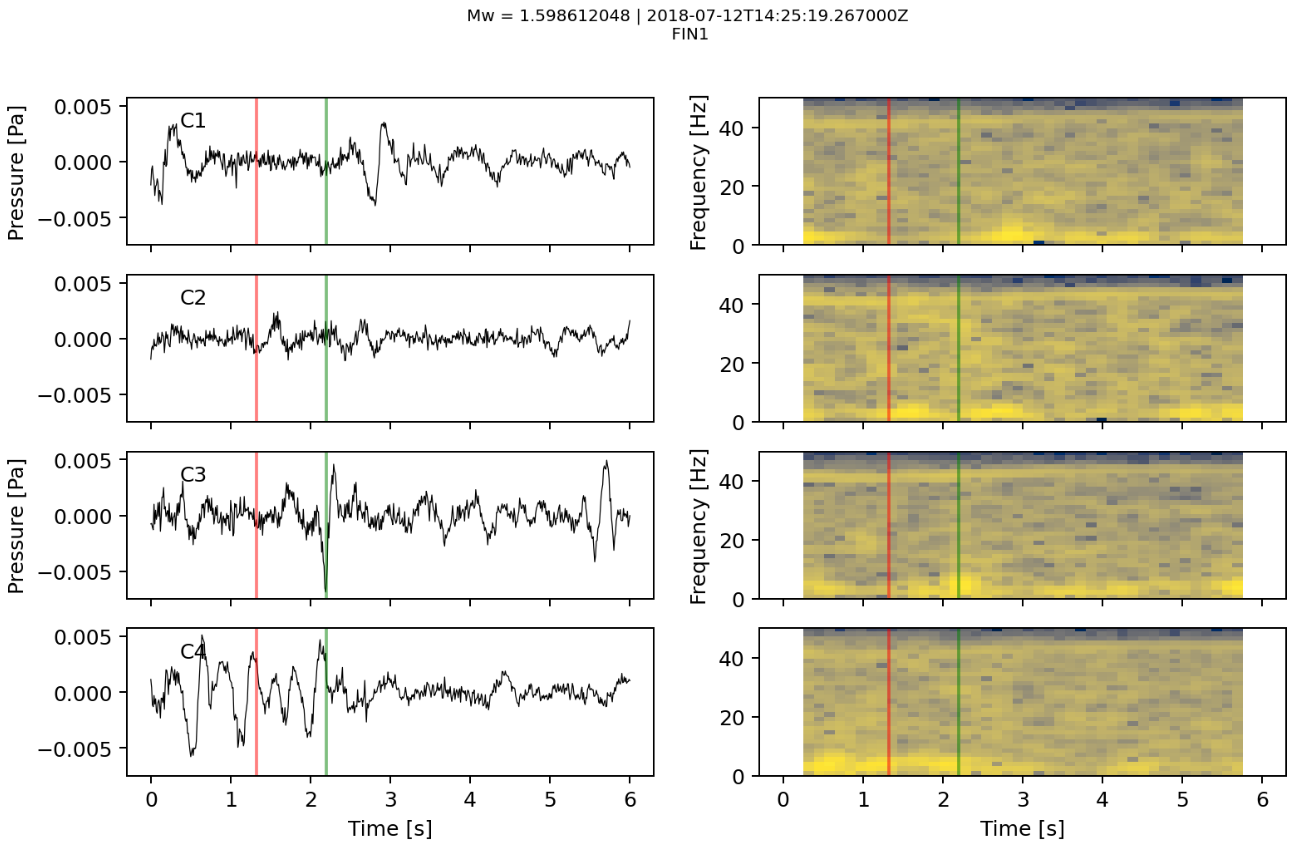

Figure S8. Waveforms (left column) and frequency spectrograms (right column) from FIN1 during a Mw 1.60 event. Red and green lines indicate estimated arrival times for P- and S-waves, respectively.

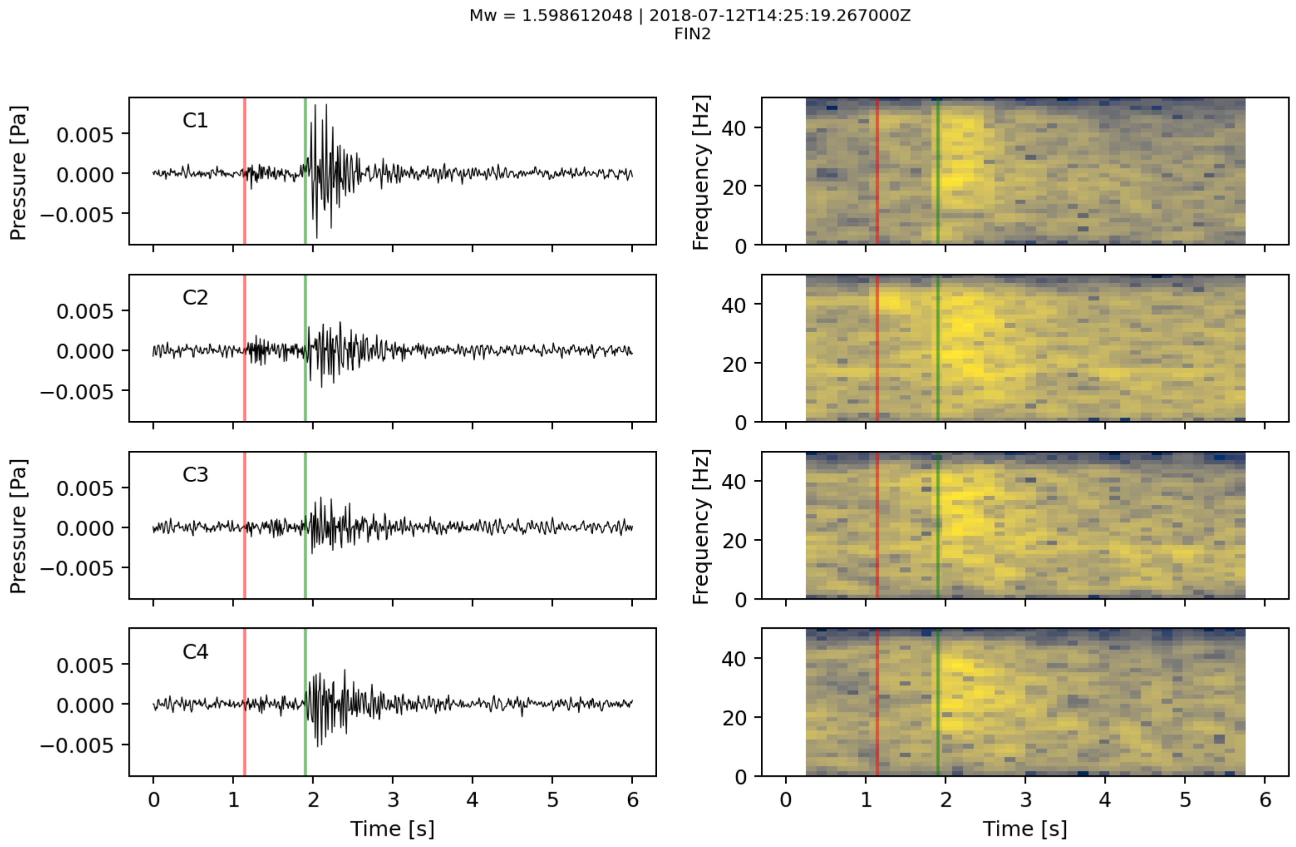

Figure S9. Waveforms (left column) and frequency spectrograms (right column) from FIN2 during a Mw 1.60 event. Red and green lines indicate estimated arrival times for P- and S-waves, respectively.

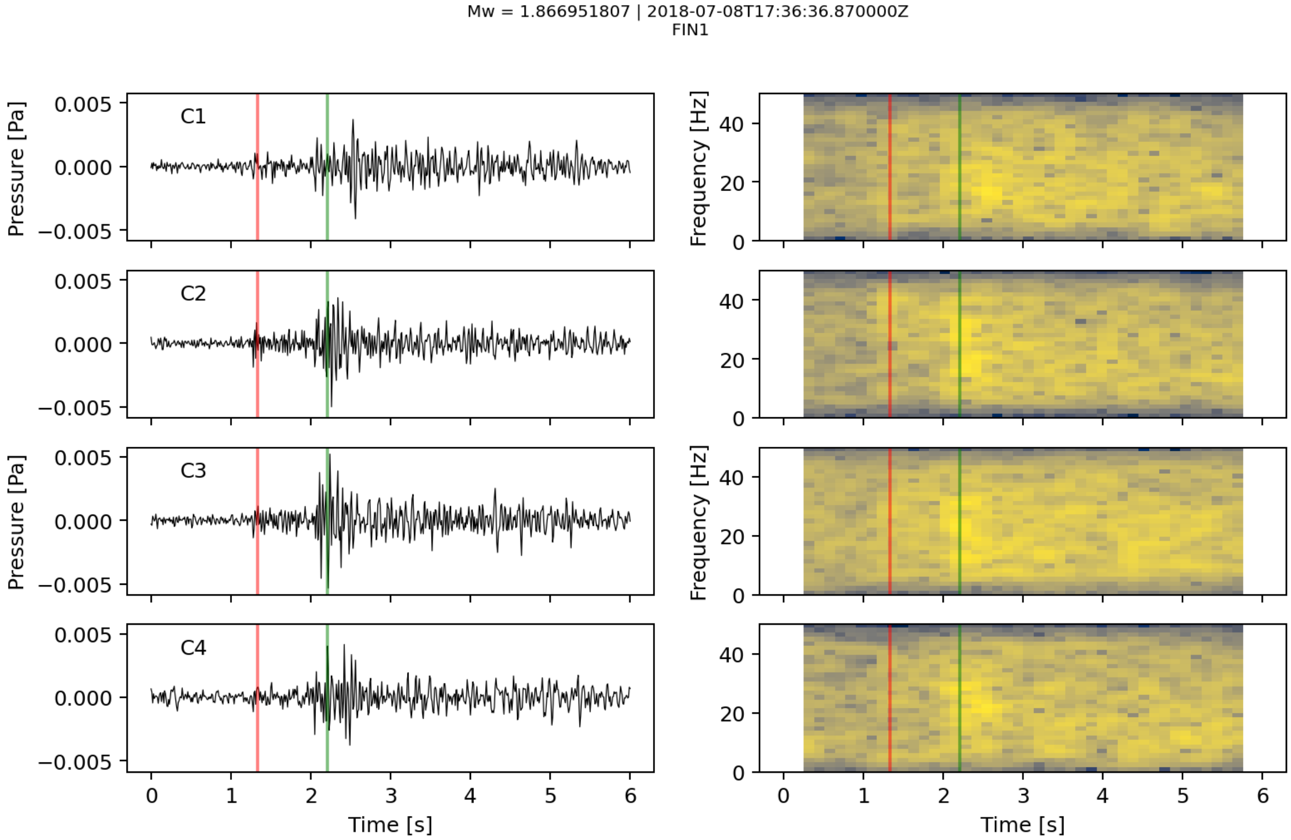

Figure S10. Waveforms (left column) and frequency spectrograms (right column) from FIN1 during a Mw 1.87 event. Red and green lines indicate estimated arrival times for P- and S-waves, respectively.

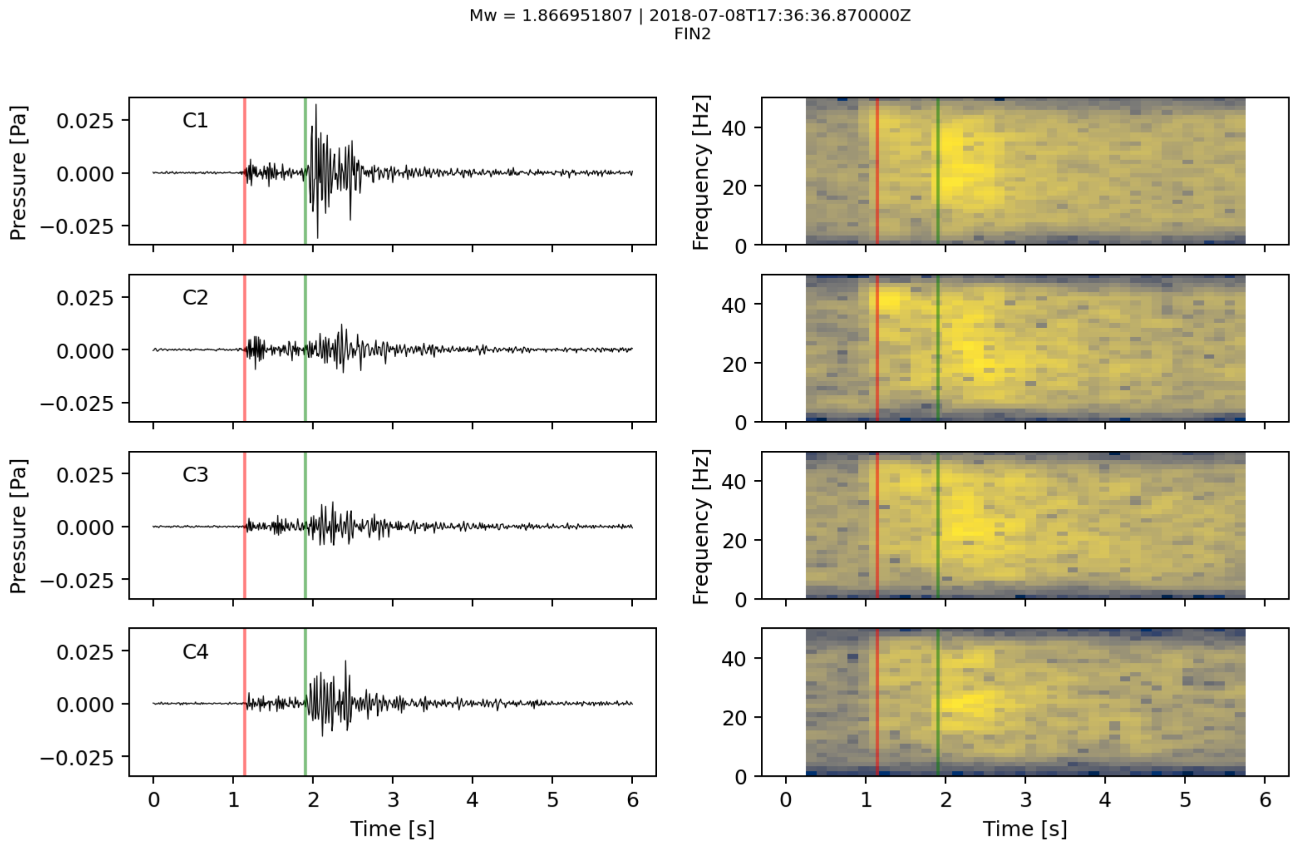

Figure S11. Waveforms (left column) and frequency spectrograms (right column) from FIN2 during a Mw 1.87 event. Red and green lines indicate estimated arrival times for P- and S-waves, respectively.

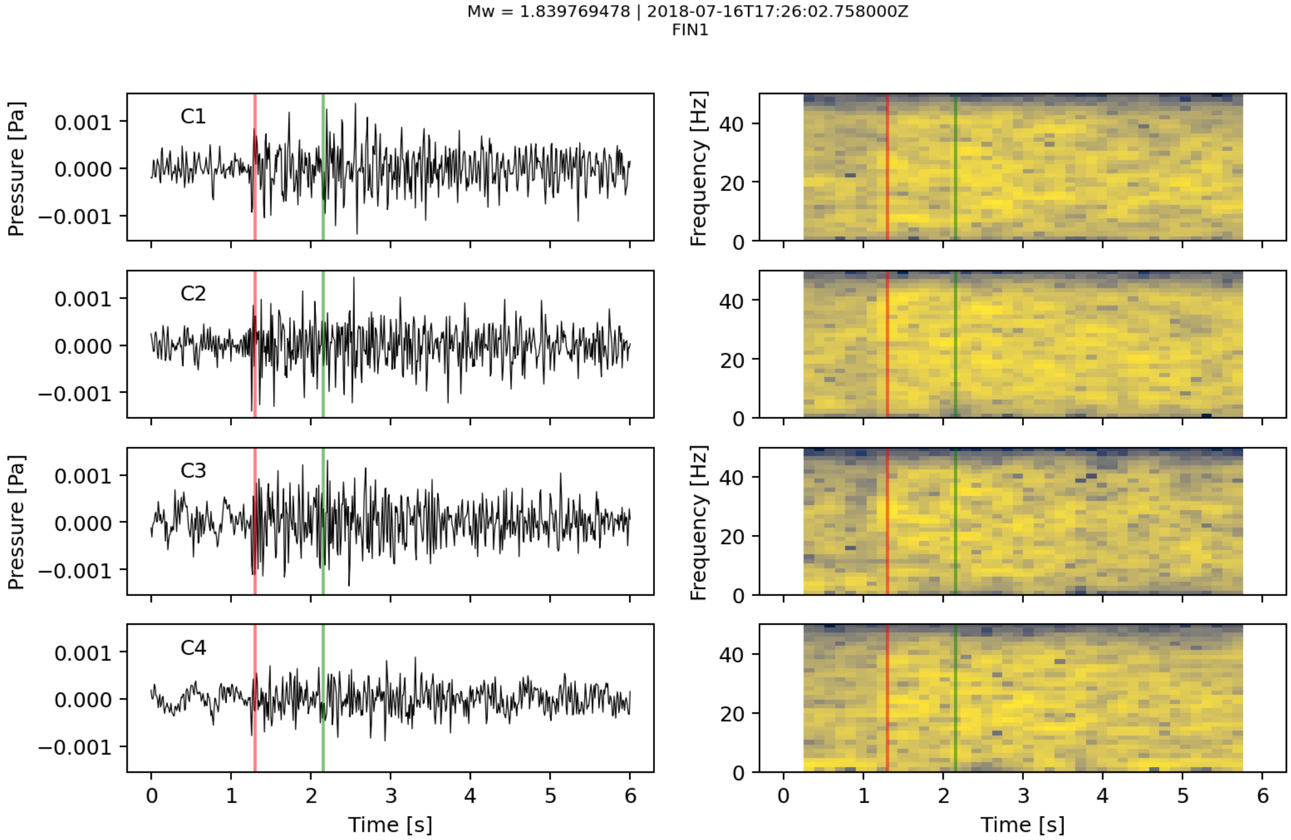

Figure S12. Waveforms (left column) and frequency spectrograms (right column) from FIN1 during a Mw 1.84 event. Red and green lines indicate estimated arrival times for P- and S-waves, respectively.

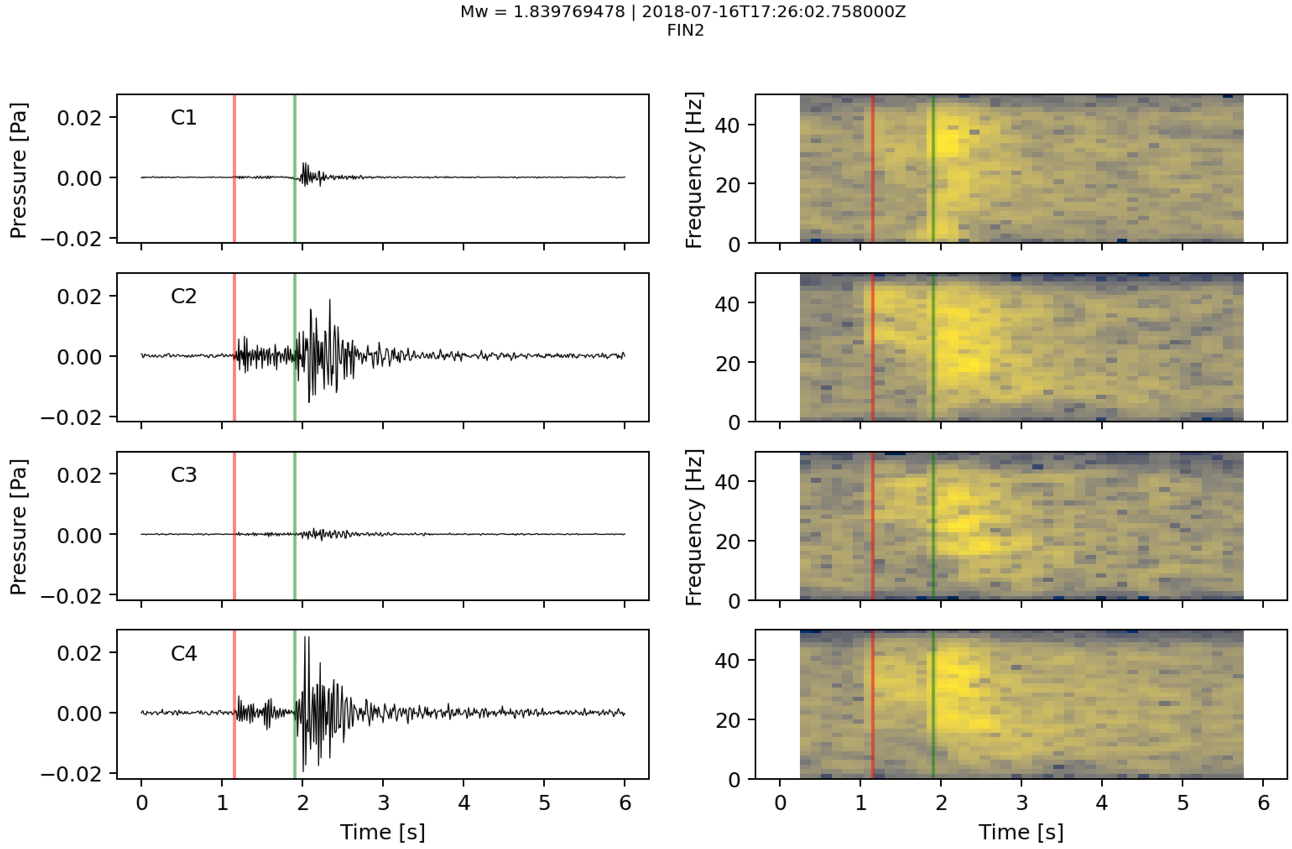

Figure S13. Waveforms (left column) and frequency spectrograms (right column) from FIN2 during a Mw 1.84 event. Red and green lines indicate estimated arrival times for P- and S-waves, respectively.

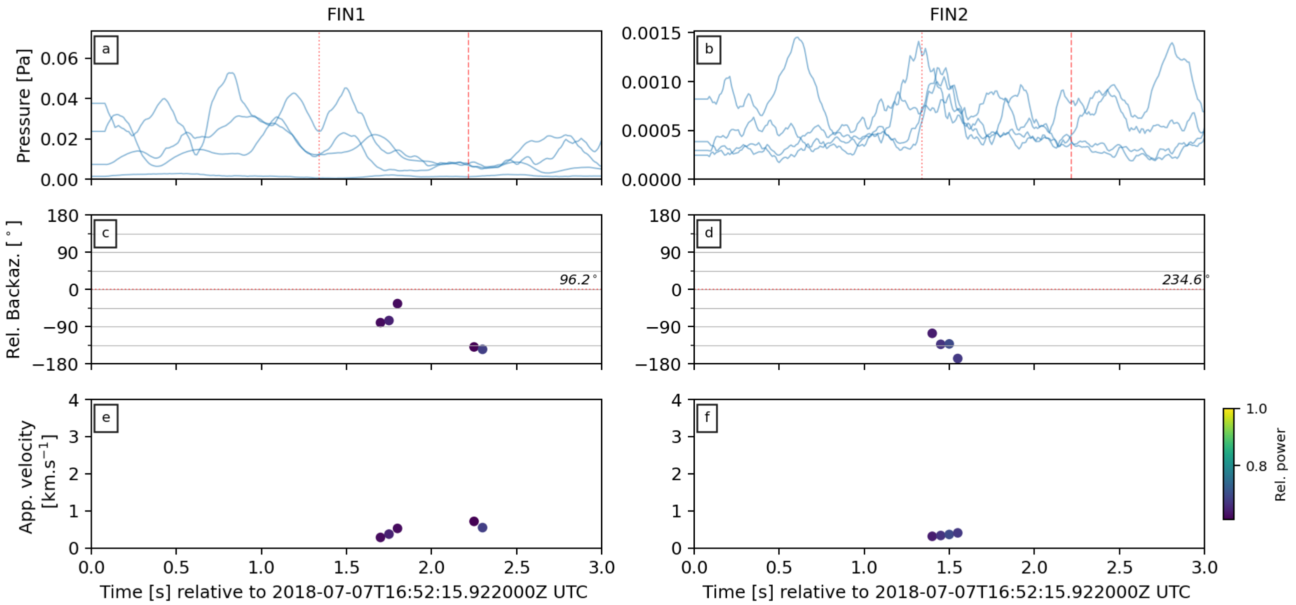

Figure S14. Beamforming results for arrays FIN1 (left column) and FIN2 (right column) for the first 3 seconds after the M_w -0.77 event. (a, b) Smoothed waveform envelopes from each element in each array. Dotted and dashed lines plot the estimated arrival times of P- and S-waves, respectively (from epicentre to array). (c, d) Back azimuth calculations for 0.5 s moving windows with 90% overlap, relative to the theoretical back azimuth from array to the M_w1.86 event epicentre (horizontal dotted line, absolute back azimuth value labeled on right hand side). (e, f) Calculated apparent velocity values across each array for each 0.5 s window. Points in panels c-f are colored by relative power, where lighter colors indicate higher relative power.

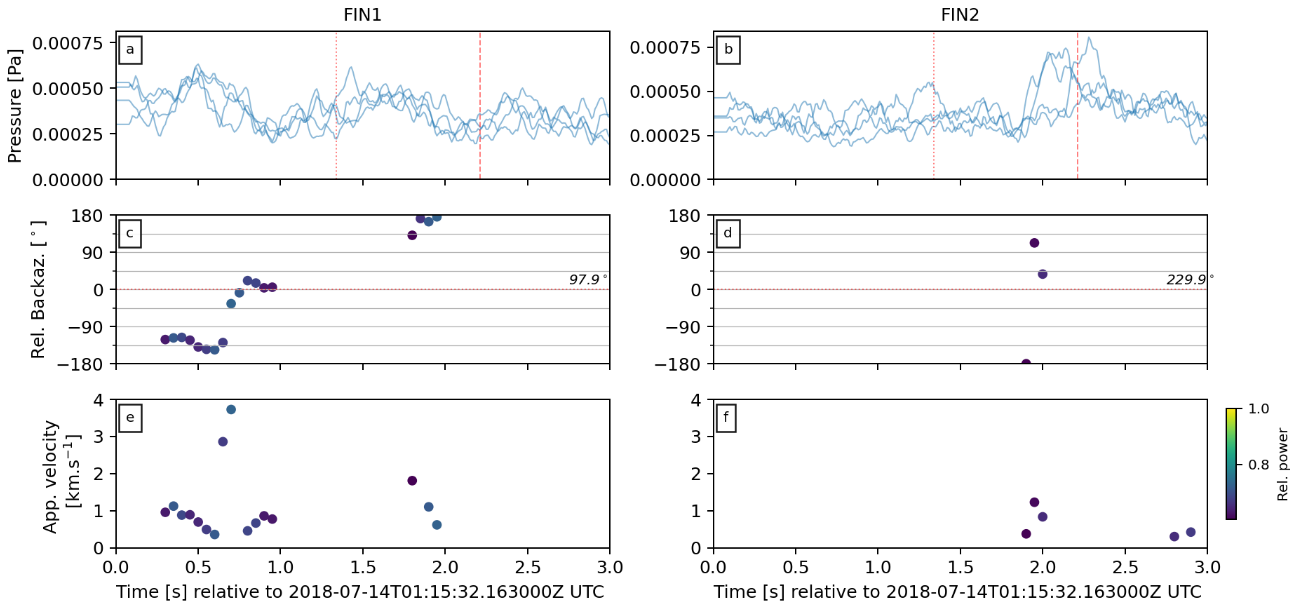

Figure S15. Beamforming results for arrays FIN1 (left column) and FIN2 (right column) for the first 3 seconds after the M_w 1.06 event. (a, b) Smoothed waveform envelopes from each element in each array. Dotted and dashed lines plot the estimated arrival times of P- and S-waves, respectively (from epicentre to array). (c, d) Back azimuth calculations for 0.5 s moving windows with 90% overlap, relative to the theoretical back azimuth from array to the M_w1.86 event epicentre (horizontal dotted line, absolute back azimuth value labeled on right hand side). (e, f) Calculated apparent velocity values across each array for each 0.5 s window. Points in panels c-f are colored by relative power, where lighter colors indicate higher relative power.

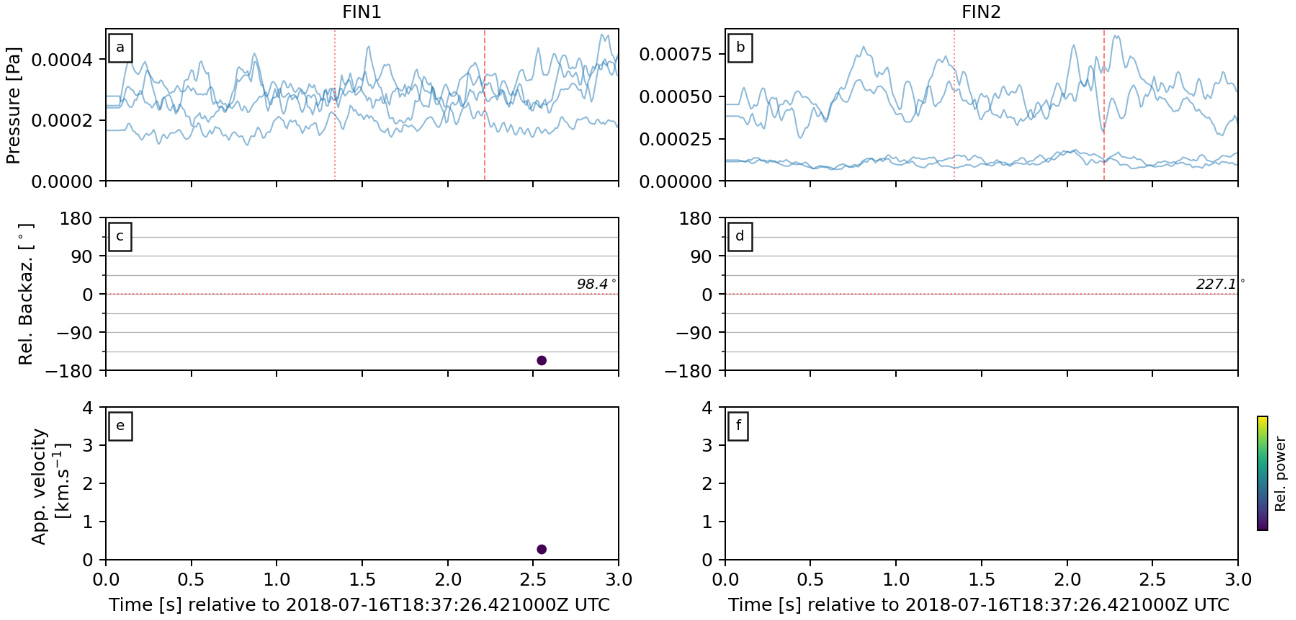

Figure S16. Beamforming results for arrays FIN1 (left column) and FIN2 (right column) for the first 3 seconds after the M_w 1.22 event. (a, b) Smoothed waveform envelopes from each element in each array. Dotted and dashed lines plot the estimated arrival times of P- and S-waves, respectively (from epicentre to array). (c, d) Back azimuth calculations for 0.5 s moving windows with 90% overlap, relative to the theoretical back azimuth from array to the M_w1.86 event epicentre (horizontal dotted line, absolute back azimuth value labeled on right hand side). (e, f) Calculated apparent velocity values across each array for each 0.5 s window. Points in panels c-f are colored by relative power, where lighter colors indicate higher relative power.

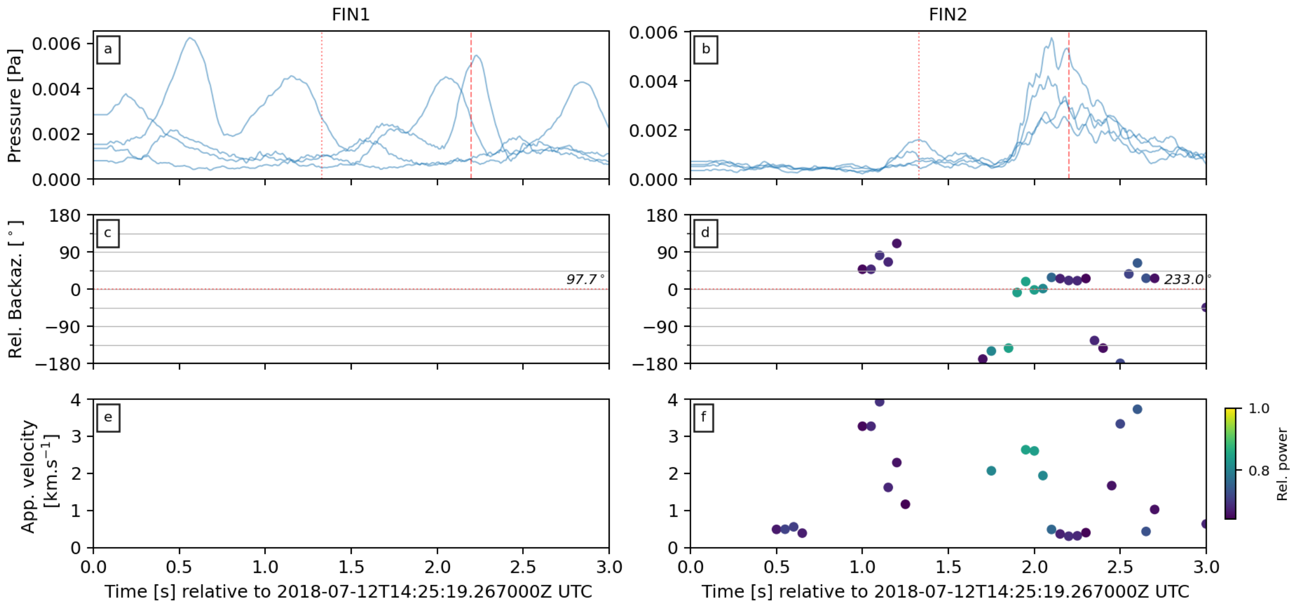

Figure S17. Beamforming results for arrays FIN1 (left column) and FIN2 (right column) for the first 3 seconds after the M_w1.60 event. (a, b) Smoothed waveform envelopes from each element in each array. Dotted and dashed lines plot the estimated arrival times of P- and S-waves, respectively (from epicentre to array). (c, d) Back azimuth calculations for 0.5 s moving windows with 90% overlap, relative to the theoretical back azimuth from array to the M_w1.86 event epicentre (horizontal dotted line, absolute back azimuth value labeled on right hand side). (e, f) Calculated apparent velocity values across each array for each 0.5 s window. Points in panels c-f are colored by relative power, where lighter colors indicate higher relative power.

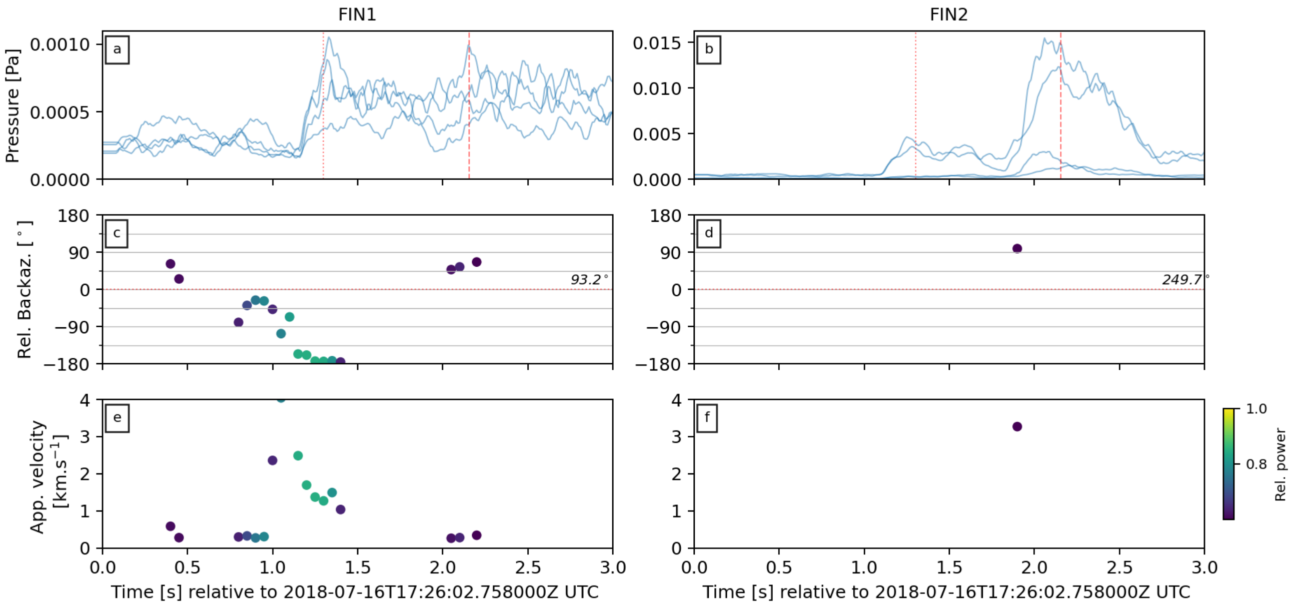

Figure S18. Beamforming results for arrays FIN1 (left column) and FIN2 (right column) for the first 3 seconds after the M_w 1.84 event. (a, b) Smoothed waveform envelopes from each element in each array. Dotted and dashed lines plot the estimated arrival times of P- and S-waves, respectively (from epicentre to array). (c, d) Back azimuth calculations for 0.5 s moving windows with 90% overlap, relative to the theoretical back azimuth from array to the M_w1.84 event epicentre (horizontal dotted line, absolute back azimuth value labeled on right hand side). (e, f) Calculated apparent velocity values across each array for each 0.5 s window. Points in panels c-f are colored by relative power, where lighter colors indicate higher relative power.

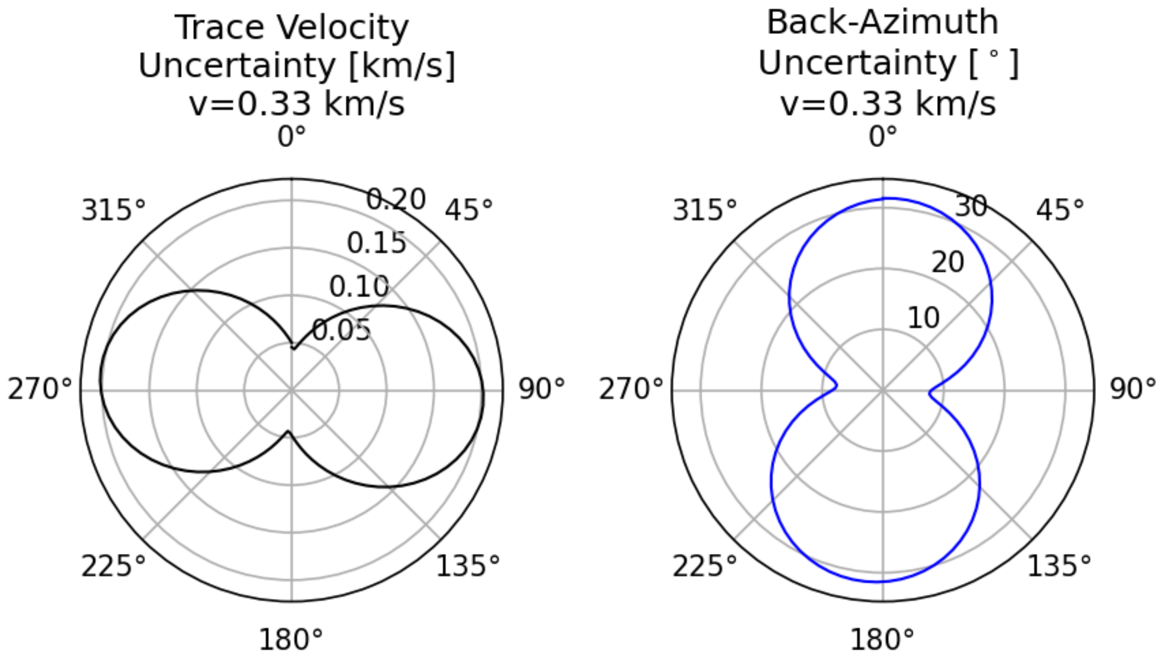

Figure S19. Rose plots highlighting the uncertainties in trace velocity (left) and back azimuth (right) in the 95% confidence interval for the FIN1 array.

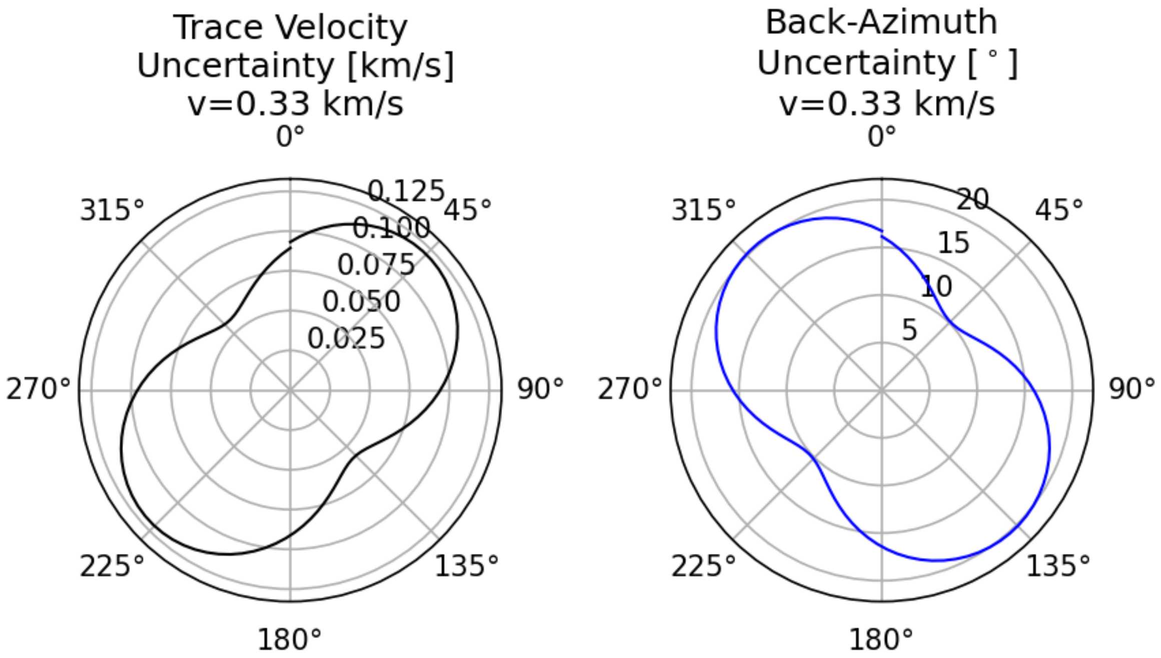

Figure S20. Rose plots highlighting the uncertainties in trace velocity (left) and back azimuth (right) in the 95% confidence interval for the FIN2 array.
